# GATA2 co-opts TGFβ1/SMAD4 oncogenic signaling and inherited variants at 6q22 to modulate prostate cancer progression

**DOI:** 10.1186/s13046-023-02745-7

**Published:** 2023-08-08

**Authors:** Xiayun Yang, Qin Zhang, Shuxuan Li, Raman Devarajan, Binjie Luo, Zenglai Tan, Zixian Wang, Nikolaos Giannareas, Tomasz Wenta, Wenlong Ma, Yuqing Li, Yuehong Yang, Aki Manninen, Song Wu, Gong-Hong Wei

**Affiliations:** 1 Disease Networks Research Unit, Faculty of Biochemistry and Molecular Medicine & Biocenter Oulu, University of Oulu, Oulu, Finland; 2 Institute of Urology, The Third Affiliated Hospital of Shenzhen University (Luohu Hospital Group), Shenzhen, China; 3 Fudan University Shanghai Cancer Center & MOE Key Laboratory of Metabolism and Molecular Medicine and Department of Biochemistry and Molecular Biology of School of Basic Medical Sciences, Shanghai Medical College of Fudan University, Shanghai, China; 4 Institute of Urology, South China Hospital of Shenzhen University, Shenzhen, China

**Keywords:** GATA2, SMAD4, TGFβ1, rs339331, Prostate cancer

## Abstract

**Background:**

Aberrant somatic genomic alteration including copy number amplification is a hallmark of cancer genomes. We previously profiled genomic landscapes of prostate cancer (PCa), yet the underlying causal genes with prognostic potential has not been defined. It remains unclear how a somatic genomic event cooperates with inherited germline variants contribute to cancer predisposition and progression.

**Methods:**

We applied integrated genomic and clinical data, experimental models and bioinformatic analysis to identify GATA2 as a highly prevalent metastasis-associated genomic amplification in PCa. Biological roles of GATA2 in PCa metastasis was determined in vitro and in vivo. Global chromatin co-occupancy and co-regulation of GATA2 and SMAD4 was investigated by coimmunoprecipitation, ChIP-seq and RNA-seq assays. Tumor cellular assays, qRT-PCR, western blot, ChIP, luciferase assays and CRISPR-Cas9 editing methods were performed to mechanistically understand the cooperation of GATA2 with SMAD4 in promoting TGFβ1 and AR signaling and mediating inherited PCa risk and progression.

**Results:**

In this study, by integrated genomics and experimental analysis, we identified GATA2 as a prevalent metastasis-associated genomic amplification to transcriptionally augment its own expression in PCa. Functional experiments demonstrated that GATA2 physically interacted and cooperated with SMAD4 for genome-wide chromatin co-occupancy and co-regulation of PCa genes and metastasis pathways like TGFβ signaling. Mechanistically, GATA2 was cooperative with SMAD4 to enhance TGFβ and AR signaling pathways, and activated the expression of TGFβ1 via directly binding to a distal enhancer of TGFβ1. Strinkingly, GATA2 and SMAD4 globally mediated inherited PCa risk and formed a transcriptional complex with HOXB13 at the PCa risk-associated rs339331/6q22 enhancer, leading to increased expression of the PCa susceptibility gene RFX6.

**Conclusions:**

Our study prioritizes causal genomic amplification genes with prognostic values in PCa and reveals the pivotal roles of GATA2 in transcriptionally activating the expression of its own and TGFβ1, thereby co-opting to TGFβ1/SMAD4 signaling and RFX6 at 6q22 to modulate PCa predisposition and progression.

**Supplementary Information:**

The online version contains supplementary material available at 10.1186/s13046-023-02745-7.

## Background

Prostate cancer (PCa) is the second most diagnosed cancer and the fifth leading cause of cancer death in men worldwide, with an estimated 1.4 million new cases and 375,000 new deaths from GLOBOCAN 2020 [1], representing a major global healthcare problem. Clinically, the diagnosis, prognosis and treatment decision of PCa are primarily made on the basis of prostate-specific antigen (PSA) level, tumor stage and Gleason scoring [2, 3], often leading to a significant overdiagnosis, overtreatment or eventually the aggressiveness of PCa [4]. Hence, there is an urgent need to identify novel precise prediction markers and therapeutic targets for PCa. Advances in next generation sequencing technologies enable examination of large scale whole-genome, whole-transcriptome and genome-wide DNA methylation data in diverse ethnic and racial cohorts, providing a foundation for better understanding and sophisticatedly identifying promising new diagnostic and therapeutic targets [5–8]. Previous studies have established that the genes within frequently amplified regions show increased expression, often playing causal roles in oncogenesis and associating with cancer relapse and metastatic progression [5, 9–12]. Recently, by integrating an analysis of whole-genome and transcriptome sequencing datasets of two PCa cohorts [8, 13] with a collected genome-wide CRISPR/Cas9-mediated loss-of-function screen data in PCa cells [14], we observed that GATA2 is top-ranked among all other copy number amplified potential causal genes, making it an interesting target to further explore its disease predictive and causal roles in PCa.

GATA2 is a member of the GATA binding transcription factor family and has been implicated in PCa progression [15]. Thus, targeting GATA2 is a highly attractive therapeutic strategy by which may improve the clinical outcome of PCa patients. However, attempt to direct targeting GATA2 is still unavailable owing to the deficiency of its biological mechanisms and 3D structure information [15], and represent as an overall difficulty in targeting transcription factors in cancer [16]. Despite of this, endeavours have been undertaken to investigate its mechanisms of function. The best characterized function of GATA2 is to exert as a pioneer transcription factor, by binding to the DNA regions of closed chromatin, initiating hierarchical recruitment and occupancy of other regulatory proteins like FOXA1 and AR to regulate genes in promoting prostate tumor growth and metastasis via AR-dependent or AR-independent network cascades [17–22]. Thus, disruption of protein–protein interaction network represents an emerging drug targets for promising therapeutic approaches in PCa [23]. However, it remains to reveal the regulatory mechanisms underlying GATA2 overexpression and the consequence in PCa as well as whether and how GATA2 is cooperating with androgen or other signaling pathways via undefined cooperating factors contributing to PCa progression.

Transforming growth factor-β (TGF-β) is well known to play double-edged sword role in cancer progression. In early stage of cancer, TGF-β acts as a tumour suppressor by inducing cell cycle arrest and apoptosis to prevent uncontrolled proliferation. In late stage of cancer, TGF-β promotes cell migration, invasion and angiogenesis, leading to tumour metastasis. These pleiotropic functions of TGF-β have been attributed to differences in the cellular context that determine TGF-β responses [24–26]. As a signal transduction downstream effector protein in TGF-β signaling, SMAD4 (Sma Mothers Against Decapentaplegia homologue 4) or DPC4 (Deleted in Pancreatic Cancer, locus 4), is a key mediator for the response to TGFβ signal by stabilizating Smad DNA-binding complexes or recruiting transcription factors and coactivators in gene expression control [27, 28], thus leading to pleiotropic roles of TGF-β in cancer progression. Previous studies suggest that SMAD4 acts as a tumor metastatic suppressor in the exquisite context of PTEN-loss mouse models for PCa [29, 30], whereas TGFβ/SMAD4 activation has also been reported to promote metastatic progression of cancers including PCa [31, 32]. Herein we proved a robust evidence in support of in vivo interaction between GATA2 and SMAD4 and thus sought to unravel the mechanistic roles of TGFβ1/SMAD4 in the GATA2-dependent context in PCa tumor progression to advanced stages and to understand how GATA2 and SMAD4 mediate inherited PCa risk.

PCa is a type of most heritable disease with both environmental and genetic factors contributing to PCa predisposition and progression. One of the best-known risk factors for PCa is familial inheritance [33], with the identification of high-penetrance rare germline mutations in BRCA1/2, CHEK2, and HOXB13 that can explain a roughly 6% of genetic predisposition of PCa [34]. Recent genome-wide association studies (GWASs) have discovered over 270 single nucleotide polymorphism (SNP) loci associated with PCa susceptibility [35–37]. By investigating the molecular mechanisms underlying the causal actions and biological effect of these risk SNPs, we and others have reported that risk SNPs often affect gene regulation by modulating chromatin binding of key transcription factors such as HOXB13, FOXA1 and AR [38–41]. Given the intertwined relationship between GATA2 and these proteins as well as herein newly-identified TGFβ1/SMAD4 signaling in the control of GATA2, we reasoned that GATA2 and its interaction factors may explain more of gene regulatory mechanisms underpinning PCa susceptibility.

In this work, we sought to identify potential causal genes in frequently amplified regions of PCa genomes that positively correlate with their expression levels and poor prognosis of PCa patients. We found that GATA2 indicates metastasis-associated copy number amplification and overexpression, and mechanistically drives its own expression via a positive feedback regulatory circuit, correlating with poor clinical outcomes in PCa and contributing to PCa cell invasion and metastasis. Intriguingly, we revealed that GATA2 interacts and cooperates with SMAD4 to promote TGFβ and AR signaling pathways and importantly, transcriptionally activates the expression of TGFβ1 by directly binding to a distal enhancer of TGFβ1. We finally found that GATA2 and SMAD4 globally mediate inherited PCa risk. These findings provide insights into further developing genetic marker GATA2 and its interaction partners or target genes to distinguishes aggressive disease and highlight the interplays between somatic genomic alterations and inherited genetic variations that are crucial for PCa progression.

## Materials and methods

### Cell culture

The LNCaP, 22Rv1, PC3, VCaP, RWPE1, RWPE2 and 293 T cell lines were originally obtained from the American Type Culture Collection (ATCC), and have been authenticated by STR profiling. The LNCaP-1F5 and V16A cell lines were kindly provided by Prof. Hansen He, University of Toronto and Prof. Olli A Jänne, University of Helsinki, respectively. All cell lines were tested regularly for mycoplasma (EZ-PCR Mycoplasma Test Kit, 20–700-20, Biological Industries). All cell lines were found to be negative of mycoplasma during our study. LNCaP, LNCaP-1F5, V16A, PC3 and 22Rv1 cells were cultured in RPMI1640 medium (Sigma), VCaP and 293T cells were grown in DMEM (Invitrogen), RWPE1 and RWPE2 cells were cultured in Keratinocyte-Serum Free Medium (KSF, Invitrogen). All mediums were supplemented with 10% FBS (Gibco) and 1% antibiotics (penicillin and streptomycine, Sigma), with the exception of KSF medium that was supplemented with the epidermal growth factor (EGF) and Bovine Pituitary Extract (BPE) included in the kit (17,005–042, Introvigen). To induce androgen signaling and AR activity, relevant PCa cells were treated with dihydrotestosterone (DHT, Sigma) for indicated time.

### Western blot assay

Cells were collected by centrifuged at 600 × *g* for 5 min and lysed on ice for 20 min using a lysis buffer (100 mM Tris–HCl, pH7.4, 2% (m/v) SDS, 10% (v/v) glycerol) containing a cocktail of protein inhibitors (Roche). Cell lysates were collected by centrifugation at 12,000 × *g* for 20 min at 4 °C and the protein concentrations were measured using the BCA protein assay kit (Thermo Scientific). Protein samples (30 μg) were separated by 8–12% SDS-PAGE and blotted onto 0.45-μm PVDF transfer membrane (Immobilon-P, Millipore) with a Semi-Dry transfer cell (Trans-Blot SD, Bio-Rad). Thereafter, the membranes were blocked with 5% non-fat milk for 1 h at room temperature and incubated with primary antibodies (1:1000 dilutions in TBST) at 4 °C overnight. The antibodies used in this study can be found in Table S1. After incubation with primary antibodies, membranes were washed three times with TBST and incubated at room temperature for 1 h with corresponding horseradish peroxidase-conjugated secondary antibodies (Santa Cruz, CA, USA) diluted at 1:5000 in TBST. Blots were detected using Pierce ECL Western Blotting Substrate. Membranes were imaged with a LAS-3000 Luminescent Image Analyzer (FujiFilm).

### Cell viability and proliferation assays

LNCaP-1F5, V16A and 22Rv1 cells were seeded in 96-well plates, respectively (5 × 10^3^ cells per well). After 48 h, 1F5 and V16A cells were transfected with control siRNAs, GATA2 or SMAD4 siRNAs together with lipofectamine RNAiMAX transfection reagent (13,778,150, Thermo Scientific). Cell viability and proliferation were determined by the cell proliferation kit II(11,465,015,001, Roche) following the manufactural instructions. Values were obtained from five replicate wells for each treatment at given time point and statistical significant was assessed using student’s *t* test. The sequences of the siRNAs were listed in Table S2.

### Wound healing assay

Cells were detached by trypsinization and resuspended into the medium supplemented with or without 1 μg/mL Dox (D9891, Sigma). Then 100 μl cell suspension containing 2 × 10^4^ cells were planted into each well of a 96-well ImageLockTM culture plate (4379, Essen BioScience). Forty-eight hours later, when the cell confluence reached to 100%, 10 μg/mL mitomycin C was pretreated with cells for 2 h. After washing with PBS, the precise and reproducible wounds in all wells were created according to the wounding procedure by using WoundMakerTM. After wounding, the media from each well were removed and the cells were gently washed two times with 1xPBS. After washing, 100 μl of medium with or without Dox were added into each well. Then the plates were placed into the IncuCyte ZOOM which the ZOOM software was set to scan the plates every 3 h.

### Transwell invasion and migration assays

Cells were pretreated with or without 1 µg/mL Dox. After 24 h treatment, cells were detached by trypsinization and resuspended into serum free or growth factor free medium. Next, 5 × 10^4^ 22Rv1, PC3, 1F5, V16A or 1 × 10^5^ RWPE2 cells in 200 μL total volume were transferred into 8-μm Transwell inserts (Corning Costar) with or without 100 µL Matrigel (Corning 350,230) coating which was diluted with serum free medium to 250 µg/mL. The lower chambers were filled with 700 µL medium containing 10% FBS. After 24 h or 48 h, cells were fixed with 4% PFA and stained with 0.02% crystal violet solution. Noninvasive or nonmigrative cells in the upper layer were removed using cotton swabs. Invasive or migrative cells on the lower side of the filters were quantified by counting the numbers of cells in eight-twelve microscopic fields (acquired at 20 × magnification) per membrane.

### Exogenous and endogenous coimmunoprecipitation (co-IP) analysis

For exogenous immunoprecipitation, 293T cells were seeded on six-well plates and cultured for 24 h. Then cells were co-transfected with pLVET-Flag-GATA2 and pLVET-V5-SMAD4 or empty vectors as control by using LipofectamineTM 3000 Transfection Reagent (L3000015, InvitrogenTM). Forty-eight hours after transfection, cell lysates were prepared using a nondenaturing lysis buffer (50 mM Tris–HCl, pH 8.0, 150 mM NaCl, 1 mM EDTA, 10% glycerol, 10% glycerol, 1 × complete cocktail protease inhibitor). For endogenous coimmunoprecipitation, LNCaP cells were seeded on 10 cm plates. After 72 h, cells were collected and prepared with the nondenaturing lysis buffer. The prepared cell lysates were incubated with 2 μl of anti-V5 or anti-Flag antibody at 4 °C overnight. Next day, 30 μl of protein G agarose beads (10004D, Invitrogen) were added to the lysate-antibody mixture and gently shaking for another 4 h. Afterwards, beads were washed three times with the lysis buffer and boiled with 2 × SDS loading buffer at 98 °C for 10 min. The prepared samples were analyzed by the western blot assay according to the instruction.

### GST-pull down assay

Briefly, the GST-SMAD4 plasmid was transformed into BL21 competence cells and cultured in Luria–Bertani (LB) with ampicillin. The corresponding fusion protein was induced by 0.5 mM isopropyl-β-D-1-thiogalactopyranoside (IPTG) at 20 °C for 16 h. The Flag-GATA2 was collected from 1F5 cells that stably overexpressed flag-GATA2. Then GST pull down assays were carried out following the instruction of PierceTM GST Protein Interaction Pull-Down Kit (Thermo Fisher Scientific, 21,516). Briefly, BL21 cells expressing GST-SMAD4 proteins were treated with pull-down lysis buffer and immobilized on equilibrated glutathione agarose resin at 4 °C for 2 h. The resin was washed with washing solution while the 1F5 lysates carrying Flag-GATA2 proteins were added with or without 250 units Benzonase per milliliter (Merk, E1014), followed by incubation at 4 °C for 14 h. After washing with a wash solution, the resin was eluted with glutathione elution buffer. The protein samples were examined by western blot.

### Chromatin immunoprecipitation (ChIP)

Cells were cross-linked in a concentration of 1% formaldehyde for 10 min at room temperature and then final concentration of 125 mM glycine was added to quench the reaction. Next, cell pellets were collected, resuspended with the hypotonic lysis buffer (10 mM KCl, 20 mM pH8.0 Tris–HCl, 10% Glycerol, 2 mM DTT) and gently rotated at 4 °C for 50 min to isolate the nuclei. The nuclei were washed with cold PBS for twice and resuspended in SDS lysis buffer (50 mM Tris–HCl, pH8.1, 10 mM EDTA, 1%SDS and 1 × Protease inhibitor cocktail (Roche)). Nuclear extracts were sonicated to generate the chromatin fragments in an average size of 250 bp using a Q800R sonicator (QSonica). For immunoprecipitation, 70 μl dynabead slurry protein G was washed twice with blocking buffer (0.5% BSA in IP buffer) and added to each reaction, followed by incubation with 6 μg indicated antibodies against GATA2, SMAD4 or corresponding control IgG at 4 °C for 12 h. After incubation, the supernatant was removed and the 250 μg soluble chromatin fragments diluted in IP buffer (20 mM Tris–HCl, pH8.0, with 2 mM EDTA, 150 mM NaCl, 1%Triton X-100, and Protease inhibitor cocktail) was added to the beads-antibody complex. After another 12 h incubation, the supernatant was removed and the DNA–protein complex precipitated with Dynabead protein G was washed 5 times with the RIPA washing buffer (50 mM pH 7.6 HEPES, 1 mM EDTA, 0.7% sodium deoxycholate, 1% NP-40, 0.5 M LiCl), followed by two times with 100 mM ammonium hydrogen carbonate (AMBIC) solution. Then the DNA–protein complex was eluted by the DNA extraction buffer (10 mM Tris–HCl, pH 8.0, 1 mM EDTA, and 1% SDS) and treated with Proteinase K and RNase A at 65 °C overnight for reverse cross-linking. The immunoprecipitated DNA was purified with the QIAquick PCR purification kit or the Mini-Elute PCR purification kit (Qiagen) and analyzed by follow-up massive parallel sequencing or qRT-PCR assay.

### Chromatin immunoprecipitation sequencing (ChIP-seq)

TruSeq ChIP Library Preparation Kit by Illumina (P-202–900, Illumina) was used to construct DNA libraries as described in the manufacturer’s instructions with a PCR of 16 cycles using the Illumina indexed library primers. NextSeq 550 was used to sequence samples (75 bp single-end) according to standard Illumina protocols. FastQC was applied to assess the quality of raw sequence reads followed by Trimmomatic [42] for quality control. The processed reads were aligned against the human genome assembly hg19 using BWA-MEM and then MACS2 [43] was applied for peak calling. UCSC associated tools [44] and Integrative Genomics Viewer (IGV) [45] were used to generate ChIP-seq signals for visualization. HOMER (v.4.1.1) [46] was used for peak annotation and differential peaks were analyzed using merged peaks with the cutoff *P*-value < 10^–4^ and fold change > 4. BEDTools [47] and Bioconductor package ChIPpeakAnno (v.3.26.2) [48] was applied to perform downstream enrichment analysis. ChIP-seq heatmaps were generated by deepTools [49].

### RNA preparation and quantitative reverse transcription PCR (qRT-PCR)

Total cellular RNA was extracted using the RNeasy MinElute Kit (74,204, Qiagen) according to the manufacture’s instruction. RT-PCR was examined using SYBR Green Master Mix (Applied Biosystems) according to the manufacturer’s procedures. The sequences of the primers used for qRT-PCR were listed in Table S3.

### RNA-sequencing (RNA-seq) and differential gene expression analysis

Total cellular RNA was extracted using the RNeasy MinElute Kit (74,204, Qiagen). The quality of RNA was validated by NanoDrop spectrophotometer and Agilent 2100 Bioanalyzer. Samples with A260/A280 and A260/A230 OD values over 2.0 and the RNA integrity numbers over 8 were used for library preparation. The library construction and sequencing were performed in the Illumina platform provided by Novogene Europe (United Kingdom). Paired-end raw sequence reads of 150 bp were first pre-examined by FastQC which is available online (https://www.bioinformatics.babraham.ac.uk/projects/fastqc/) for quality assessment. Trimmomatic was then applied for quality control, followed by a final FastQC run on cleaned reads to ensure read quality. STAR version 2.7.2a [50] was employed to map processed reads to the human genome hg38 by default settings and aligned reads were then quantitated by HTSeq [51] and gene annotation from Encode with parameters “-s no, -I gene_name”. Genes with low expressions (< 2 cumulative read count across samples) were filtered out prior to the differential expression analysis using DESeq2 (1.26.0) [52]. Differentially expressed genes were identified by cutoff FDR < 0.1. Data was normalized using Variance Stabilizing Transformation (VST) from DESeq2 and heatmaps displaying gene expression levels were plotted using R package “pheatmap” (1.0.12).

### Gene Set Enrichment Analysis (GSEA)

GSEA [53] was used to perform functional annotation of gene expression profiles in MSigDB [54] database. Differentially expressed genes were sorted in a descending order by “stat” statistics to generate the pre-ranked gene list for the GSEAPreranked test. Parameters were set as followed: Enrichment statistic = “weight”, Max size (exclude larger sets) = 5000, number of permutations = 1000, while all other variables were set as default. The GSEA enrichment plots were produced by R packages “clusterProfiler” (3.14.3) [55] and “enrichplot” (1.6.1).

### Development of the GATA2 & SMAD4 joint direct target signature

We defined the GATA2 & SMAD4 joint direct target signature by first extracting a list of common differentially dysregulated genes upon siRNA-mediated knockdown of GATA2 or SMAD4 in 1F5 cells with FDR < 0.1. We next determined the overlap of these genes with joint GATA2 and SMAD4 genome-wide chromatin binding sites from the 1F5 cells. We finally devised the joint direct target gene signature that displayed both GATA2 and SMAD4 chromatin binding in the promoter (TSS ± 5 kb) as well as dysregulation upon RNA-seq profiling of GATA2 and SMAD4 in 1F5 cells. The joint direct target signature score was calculated as weighted sum of normalized gene expression levels.

### Development of the GATA2 and/or SMAD4 eQTL gene signatures

We derived the eQTL gene signatures by incorporating GWAS associations, GATA2 or/and SMAD4 ChIP-seq profiling data. We imputed proxy SNPs based on specific ethnics data of the 1000 genome project in linkage disequilibrium (LD, R^2^ ≥ 0.5) with PCa GWAS variants. For the GATA2 eQTL gene signature, we integrated GATA2 ChIP-seq data from multiple PCa cell lines from our study and Cistrome DB to capture more enriched SNPs. For the SMAD4 eQTL gene signature, we applied SMAD4 ChIP-seq profiling data in 1F5 cells from our study due to the lack of additional online resources. To equilibrate the ChIP-seq data, we utilized GATA2 and SMAD4 ChIP-seq profiling in 1F5 cells to develop the GATA2 & SMAD4 joint eQTL gene signature. BEDTools were applied to obtain the intersected binding regions between GATA2 and SMAD4. We then calculated the amount of SNPs enriched in the chromatin binding sites of GATA2 or SMAD4 alone or their common ChIP-seq regions, and examined the SNP-gene expression association of enriched SNPs from GTEx, PancanQTL and ncRNA-eQTL.

### Motif analysis

The effect of rs339331 on transcription factor binding motifs was analyzed using R package atSNP v1.8.0 [56] (affinity test for regulatory SNP detection) in R (v. 4.1.0). Binding affinity tests were performed for the motif matches between GATA2 and rs339331 variants using the ENCODE motif library [57].

### Survival analysis

Kaplan–Meier survival analysis was applied to assess the impact of GATA2 amplification or high expression levels of GATA2 on PCa prognosis in multiple independent cohorts. Patients were first stratified into two groups based on GATA2 genomic copy number alterations or the median expression levels. For the investigation of the synergistic effect of GATA2 and RFX6 on patient survival, we excluded PCa patients harboring *RFX6* deep loss due to *RFX6* located in relatively high genomic deletion region in PCa despite that *RFX6* deletion status was reported not as a potential confounding variable accounting for the observed correlations between *RFX6* expression and clinical severity from our previous study [40]. For the examination of rs339331 genotype on PCa patient survival, we first stratified PCa tumors expressing high- or low- levels of *RFX6* and examined the prognostic value of rs339331 in these two groups separately. Note that PCa cases with *RFX6* deletion were excluded from the analysis. Kaplan–Meier survival analysis were conducted using R package “Survival” (v. 3.2.3) and assessed by using log-rank test. Cox proportional hazards model was applied to calculate the hazard ratio (HR) for assessing the relative risk between different patient groups.

### Gene expression correlation analysis

The co-expression analysis was applied in various scenarios including expression levels of GATA2 & SMAD4, GATA2 & SMAD4 joint direct target gene signature, TGFβ signaling or AR signaling by Pearson's product-moment correlation. The gene expression values from gene signatures were calculated as sums of z-score normalization. The AR signaling signature was devised with a panel of 30 representative genes from the literature [58], including *MPHOSPH9*, *ADAM7*, *FOLH1*, *CD200*, *FKBP5*, *GLRA2*, *NDRG1*, *CAMKK2*, *MAN1A1*, *MED28*, *ELL2*, *ACSL3*, *PMEPA1*, *GNMT*, *ABCC4*, *HERC3*, *PIP4K2B*, *KLK3*, *EAF2*, *CENPN*, *MAPRE2*, *NKX3.1*, *KLK2*, *AR*, *TNK1*, *MAF*, *C1ORF116*, *TMPRSS2*, *TBC1D9B* and *ZBTB10*. The TGFβ signaling signature was referenced from the MSigDB gene set Reactome signaling by TGFβ family members. The EMT score, composed of 76 genes, was referenced from Bayer et al. [59], from which the authors develop and validate a robust EMT signature by integrating gene expression, proteomic, and drug response analysis using cell lines and tumors from patients with non-small cell lung carcinoma (NSCLC). In their study, the expression levels of the 76 genes in the EMT signature are found correlated with known EMT markers. The TGFβ signaling signature and the EMT scores were calculated by the z-scored sum of gene expressions. For the investigation of the expression correlation between RFX6 and GATA2, the PCa patients with RFX6 deep loss were ruled out.

### siRNA transfections

A set of four siRNAs (Qiagen) independently against GATA2 or SMAD4 were tested by qRT-PCR. The most two efficient ones against GATA2 and SMAD4 were used for the further experiments. Cells were seeded in 96-well plates (3 × 10^5^/well) and 6-well plates (8 × 10^3^/well). After 48 h, cells were transfected with 50 nM siRNA separately target on GATA2 and SMAD4, in parallel control siRNAs using the lipofectamine RNAiMAX transfection reagent according to the instructions.

### Allele-specific quantitative RT-PCR (AS-qPCR)

AS-qPCR was performed as previous instruction [34]. In brief, the primers for allele-specific amplification of the rs339331 region with a T or C in the DNA samples from ChIP were designed as listed in Table S4.

### Lentiviral construction, lentivirus production and infection

GATA2 and SMAD4 were separately cloned from cDNA of HEK293T cells using the primers listed in Table S5. Then the pLVX-TetOne-GATA2 and pLVX-TetOne-SMAD4 constructs were generated using the standard molecular biology techniques. The shRNA constructs targeting GATA2, SMAD4 and HOXB13 in the pLKO.1-puro vector were obtained from the Functional Genomic Unit of the University of Helsinki. The information of the shRNA constructs which were used in this study could be found in Table S2. The lentivirus was produced with the third-generation packaging system in 293 T cells. Briefly, cells were seeded in 10-cm plates, after 24 h, cells reached 70–80% confluence and co-transfected with indicated overexpression constructs or shRNA constructs (6 μg each), pVSVG (envelope plasmid, 2 μg), pRSV-Rev (packaging plasmid, 2 μg) and pMDLg/pRRE (packaging plasmid, 2 μg) plasmids accompany with 36 μl Lipofectamine 2000 (Invitrogen) following the manufacturer’s instruction. Twenty-four hours after the transfection, medium was changed into the low-glucose DMEM GlutaMAX (Gibco) supplemented with 10% FBS and 1% streptomycin/penicillin. Then virus supernatant was collected every 24 h up to three days. The collected supernatant containing virus was filtered through an 0.45 μM filter unit (Millipore) and then used for cell infection. The remaining virus was divided into aliquots and stored at -80℃. For lentivirus infection, cells were split and seeded into 6-well plate. When cells were grown into 50% confluency, cells were incubated with the medium containing the indicated virus together with 8 μg/mL polybrene (Sigma). After eight hours, the medium containing the virus was removed and replaced with the fresh medium for another 24 h. Then, medium was changed into the new one with 2 μg/mL puromycin (Sigma). After 3 days, when the control cells were all dead, the surviving cells were maintained with the puromycin for another 3 days. Finally, cells were collected for the follow-up qRT-PCR, western blot and ChIP experiments.

### Ectopic expression of GATA2 in the enhancer knockout clones and control cells

Firstly, using lentivirus system as mentioned above, the pLVX-TetOne-GATA2 was stably expressed in the enhancer knockout clones and control cells. The expression of GATA2 was turn-on/off by Dox (1 μg/ml). Then total RNA was extracted and qRT-PCR was used to confirm the expression of endogenous GATA2 by primer pair that specifically crossed the exon 5 region and intron 4 region of GATA2. The primers were listed in Table S3.

### Luciferase report assay

GATA2 and SMAD4 were separately cloned into the PLVET expression plasmid and the PLVET-GATA2 and PLVET-SMAD4 constructs were established. The SBE4-Luc (16,495) and pGL3-TGFβ1 promoter construct (101,762) were purchased from Addgene. The Renilla control plasmid pGL4.75 (hRluc/CMV) was originally purchased from Promega. For plasmid transfection, V16A cells were reverse transfected with the indicated luciferase reporter plasmids using the X-tremegene HP DNA Transfection Reagent (Roche) according to the manufacturer’s instruction. Forty-eight hours later, Dual-Glo Luciferase Assay System Kit (Promega) was used to examine the luciferase activity of the transfected V16A cells. All data were obtained from five replicates and statistical analysis was performed with a two-tailed student’s test.

### CRISPR/Cas9-mediated genomic deletion of enhancer region

Two pairs of sgRNAs were designed using the benchling CRISPR design software (https://www.benchling.com/crispr/). Then the sgRNA oligos were annealed and inserted into the plasmid pSpCas9n (BB)-2A-Puro (PX462) V2.0 (a gift from Feng Zhang Lab at MIT). For transfection, 22Rv1 cells were planted into the 24-well plate and grown to 70% confluency. The total 1 μg sgRNA plasmid pair (0.5 μg each sgRNA) were transfected into the cells together with the Lipofectamine 3000 (Invitrogen). After 48 h, 2 μg/mL puromycin was added to select the puromycin positive cells. Three days later, after the control cells (non-transfected cells) were all dead, the remaining cells were collected and isolated for single clonal cells by serial dilution into 96-well plates. After 3 weeks, the single clonal cells were screened and genotyped. Then the correct enhancer knockout clones were picked up and expanded culturing for the following qRT-PCR and western blot experiments. The sequences of gRNAs used in this study were listed in Table S6.

### Quantitative analysis of chromosome conformation capture assay (3C-qPCR)

3C-qPCR was performed as previously described [39]. In brief, 1 × 10^7^ 22Rv1 cells were collected and crosslinked with 10 mL PBS which contains 1% formaldehyde at room temperature for 10 min. Then final 0.125 M of glycine was added to quench the crosslinking reaction. After quenching, cells were washed with PBS twice and resuspended with 5 mL lysis buffer (10 mM Tris–HCl, pH 7.5; 10 mM NaCl; 0.2% NP-40; 1 × complete protease inhibitor). After incubation on ice for 10 min, the lysis buffer was removed and the nuclei were washed with cold PBS twice. Then prepared chromatin was digested by HindIII overnight and the digestion efficiency was checked. The well-digested samples (the efficiency of restriction digestion was over 80%) are subjected to the follow-up ligation step. After digestion and reverse crosslinking, DNA was purified and dissolved into 150 μl of 10 mM pH 7.5 Tris. For qPCR analysis of the 3C DNA template, the sample purity was verified and the standard curves were built using the control DNA template. The values of the 3C-qPCR were required according to the intercept and slope values from the standard curves and finally normalized to the loading control ERCC3. All the primers used in this experiment were listed in Table S7.

### In vivo* animal experiments*

For metastasis, RWPE2-GATA2 and PC3-GATA2 overexpression stable cells were cultured and harvested. The ready-to-use cells were resuspended with PBS. ICR SCID male mice with 8 weeks old in each group (5 mice/group) received tail vain injection of 1 × 10^6^ cells/200 μl or left ventricle injection of 5 × 10^5^ cells/100 μl into each mouse. For inducing the expression of GATA2, the mice were given the Dox (2 mg/ml) together with the drinking water. To improve the palatability, sucrose (50 mg/ml) were added into the drinking water at the same time. Metastatic signals were observed using the IVIS system (Xenogen) with excitation and emission wavelengths at 570 nm and 620 nm, respectively. The mice were sacrificed at 4 weeks after injection. The lungs and bones were removed and fixed in paraformaldehyde (PFA).

For subcutaneous tumor model, the 22Rv1 cells transducted with shRNA scramble or shRNA against GATA2 (5 × 10^6^ cells with100 μl PBS and 100 μl Matrigel per mouse) were subcutaneously injected into the right flank of SCID mice. Ten days after inoculation, the tumor volume was measured 2 times per week. At the end of the experiment the mice were sacrificed and the tumors were removed, weighed, and fixed in paraformaldehyde (PFA).

The animal procedures were performed following the guidelines of the International Animal Care and Use Committee. Mouse models and protocols for cancer research are approved by the National Animal Experiment Board (ESAVI/3901/2021). Overall, in the animal work, the principle of 3R (reduction, refinement, replacement) is respected followed.

### Statistical analysis and data visualization

All statistical analyses were performed using RStudio (v. 1.4.1106) with R version v. 4.1.0. Statistical tests applied across normal prostate, primary tumor and metastatic tissues were assessed by the Mann–Whitney U test or the Kruskal–Wallis H test in accordance with the number of comparison groups. The association between GATA2 amplification and clinical variables were assessed by Fisher’s exact test. For the results from microarray-based expression profiling, gene probes with lowest *P* values were selected. Samples with missing expression or patient survival data were excluded from analyses. Circos maps were generated using BioCircos (v.0.3.4) [60]. *P* value < 0.05 was considered to be statistically significant. Asterisks indicate the significance level (*P < 0.05; **P < 0.01; ***P < 0.001). For experimental part, data were presented as means ± SEM and statistically analyzed by unpaired *t*-test between two groups using the GraphPad Prism 6 software. A *P* < 0.05 was considered as the significant difference.

## Results

### GATA2 amplification and upregulation correlate with PCa metastasis

**Fig. 1:**
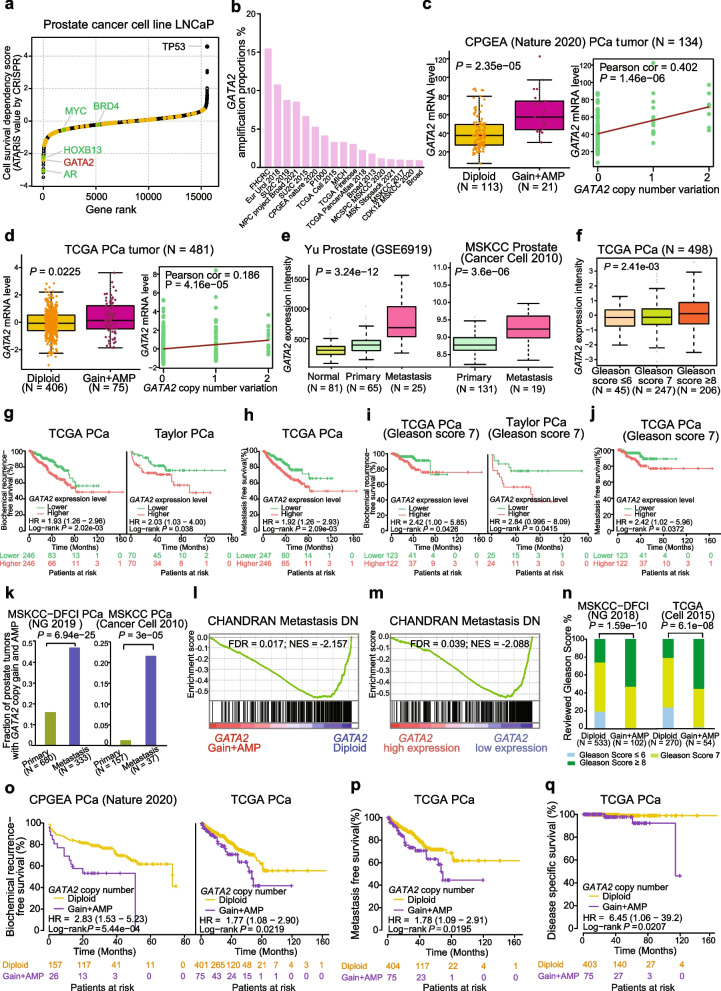
GATA2 genomic amplification and upregulation are correlated with tumor progression and poor prognosis in PCa patients. **a** Genome-wide CRISPR loss-of-function identification of the essential genes for cell survival in PCa cell LNCaP. Lower ATARiS scores indicated higher essentiality of the indicated genes for cell growth and survival. Orange dots represented causal amplification genes defined by their significant positive linear correlations between copy number gain and expression levels in the CPGEA or EU PCa cohort. Green dots highlighted *AR*, *HOXB13*, *MYC* and *BRD4* that are known to be crucial for PCa cell proliferation and survival whereas *TP53* is not favorable for PCa cell growth and survival. **b** Proportion of GATA2 genomic amplificaiton across 17 cohorts of PCa tumors in different populations. **c-d** GATA2 expression in prostate tumor tissues with GATA2 diploid or copy number gain or amplification (left panel), and Pearson correlation between GATA2 mRNA expression and copy number changes (right panel). Gain: with presence of one copy; AMP, amplification: with the presence of two copies. *P* values determined by the Mann–Whitney U test or Pearson correlaiton. AMP, amplification. **e** Box plots showing GATA2 upregulation in human primary and metastasis PCa. *P* values determined by the Kruskal–Wallis H test or the Mann–Whitney U test. **f** Elevated GATA2 expression correlated with higher Gleason score. *P* values determined by the Kruskal–Wallis H test. **g-h** Kaplan–Meier plots indicated increased biochemical recurrence and metastasis risks of PCa patients with tumors expressing higher GATA2 levels in two independent cohorts. Patient groups stratified by the median value of GATA2 expression levels. *P* values assessed by a log-rank test. **i-j** Higher expression levels of GATA2 exhibited predictive values for biochemical relapse and metastasis in PCa patient group with an intermediate risk (Gleason Score 7). *P* values assessed by a log-rank test. **k** Fraction of PCa tumors harboring GATA2 copy number gain is elevated in metastasis than in primary PCa in multiple independent cohorts of PCa patients. *P* values were examined by the Fisher's exact test. **l**-**m** Top gene set depleted in PCa tumors with GATA2 copy number gain/amplification vs diploid (**l**) or with GATA2 high (higher than the 50th percentile) versus low (lower than the 50th percentile) mRNA abundance (**m**) in the TCGA PCa cohort. NES, normalized enrichment score. FDR values calculated by the GSEA analysis. **n** Gleason scores were higher in PCa patient group with GATA2 copy gain in multiple independent PCa datasets. *P* values examined by the Fisher’s exact test. **o-q** PCa patients with GATA2 copy gain were associated with elevated risks for biochemical relapse (**o**), metastasis (**p**), and decreased disease-specific survival (**q**). *P* values assessed by a log-rank test

We previously reported a system identification of genomic alterations including a number of copy number amplified regions in PCa patients, based on an integrated analysis of whole-genome and transcriptome sequencing datasets of our PCa cohorts [8, 13], yet the relation between gene copy number change and expression remains to be fully elucidated. Studies have suggested that the genes within frequently amplified regions in the cancer genomes show increased expression levels, often playing causal roles in oncogenesis [11–13]. We thus aggregated a list of genes within these reported amplified regions in at least 5% of tumors and the RNA-seq profiling data through bioinformatic mining of the two PCa genomic datasets [8, 12] to identify potential causal amplified genes, resulting in 58 genes that showed positive correlations between their copy gain and elevated expression levels in tumors of patient with PCa (Table S8). To investigate whether the amplified potential causal genes possess clinical impact, we conducted a Kaplan–Meier estimator analysis and found that PCa patients with higher expression levels of the gene set were associated with elevated risk of biochemical recurrence and metastasis, respectively (Fig. S1a, b). We next integrated a set of genome-wide CRISPR/Cas9-mediated loss-of-function screen data in the PCa cell line LNCaP [14] and observed that GATA2, coding a known transcription factor is top-ranked among all other copy number amplified potential causal genes, displaying strongest essentiality for PCa cell survival (Fig. 1a, Fig. S1c and Table S9). It was previously reported that GATA2 was consistently amplified in human tumors, mouse models of cancer, and the mouse embryo fibroblasts (MEF) immortalization system [61]. Notably, we examined GATA2 amplification status in different ethnic populations by incorporating [17] independent PCa genomic datasets, and found that GATA2 amplification is highly prevalent across these studies (Fig. 1b).

To assess whether GATA2 genome alterations display any clinical impacts on PCa, we evaluated potential correlation between GATA2 copy number gain and its expression levels in human prostate tumors. The results showed that GATA2 expression levels are higher in patient group with GATA2 copy number gain in two independent clinical PCa datasets (Fig. 1c, d and Fig. S1d), consistent with previous notion that the genes with copy number amplification in cancer genome often show increased expression levels leading to altered activity in tumor cell growth and progression [11, 12]. In contrast, this correlation was not observed in the matched adjacent normal prostate tissues (Fig. S1e, f). Furthermore, high expression levels of GATA2 in PCa displayed highly robust associations with tumor progression to metastasis (Fig. 1e and Fig. S1g), higher Gleason score (Fig. 1f and Fig. S1h), advanced tumor stage (Fig. S1i) and elevated PSA level (Fig. S1j). In line with these observations, we also found that PCa patients with higher GATA2 expression levels were associated with elevated risk of biochemical recurrence and metastasis (Fig. 1g, h), consistent with previous notion that GATA2 upregulation or increased transcriptional activity is positively associated with poor prognosis of PCa patients [20, 62, 63]. To further explore whether GATA2 expression levels possess predictive value for PCa low- and high-risk cases, we stratified two large cohorts of PCa patients based on Gleason scores and examined potential correlation between GATA2 expression and disease severity. This analysis suggested an explicit predictive value of GATA2 mRNA levels for biochemical recurrence and metastasis in PCa patients with a Gleason score of 7 (intermediate risk, Fig. 1i, j), but not for the low-risk cases with Gleason score ≤ 6 (Fig. S1k, i) or high-risk cases with Gleason score ≥ 8 (Fig. S1m, n). These results indicate GATA2 as a potential independent prognostic marker in distinguishing PCa patients that may recur in the intermediate-risk cases who are the most difficult ones to avoid overtreatment when considering active surveillance or immediately determined therapy in clinic.

We next investigated whether GATA2 amplification correlates with PCa metastasis and thus examined the proportion of GATA2 copy number gain in PCa patients with primary and metastatic tumors in multiple independent datasets. The results showed that GATA2 amplification was tremendously more frequent in patients with metastasis than PCa primary tumors (Fig. 1k and Fig. S1o). Robust association of GATA2 amplification and upregulation with metastasis in PCa indicates its function in PCa tumorigenesis and tumor progression. We thus performed gene expression profile comparison of the TCGA PCa cohort with or without GATA2 gain/amplification using gene set enrichment analysis (GSEA). The results showed that the “CHANDRAN Metastasis DN” gene signature (genes downregulated in metastatic vs nonmetastatic prostate carcinoma) was the most enriched gene set across all gene sets depleted in PCa tumors with GATA2 gain/amplification in comparison with those GATA2 diploid tumors (Fig. 1l). In line with this, the same gene signature was found to be significantly depleted in PCa tumors expressing high mRNA levels of GATA2 (Fig. 1m), further indicating the function of GATA2 in regulating PCa metastasis.

Next, to assess clinical impact of GATA2 copy number alterations on human PCa progression, we first examined potential correlation of GATA2 copy number gain with PCa clinical variables in multiple independent datasets. The results revealed that patient group with GATA2 copy number gain was greatly associated with higher Gleason score (Fig. 1n and Fig. S1p), advanced tumor stage (Fig. S1q), elevated PSA levels (Fig. S1r) and lymph nodes (Fig. S1s). We next performed the Kaplan–Meier survival assessment to investigate the association of GATA2 copy gain and patient prognosis. The results consistently demonstrated that PCa patients with GATA2 copy gain indicates significantly increased frequency of biochemical relapse, metastasis and shorter disease-specific survival in two different PCa cohorts (Fig. 1o-q). Taken together, these data demonstrate that GATA2 has high frequent genomic amplification correlating with its increased expression, and GATA2 alterations including amplification and overexpression are profoundly associated with PCa progression to metastasis and hold potential prognostic value in PCa risk prediction.

### GATA2 drives its own expression via a positive feedback regulatory circuit

**Fig. 2:**
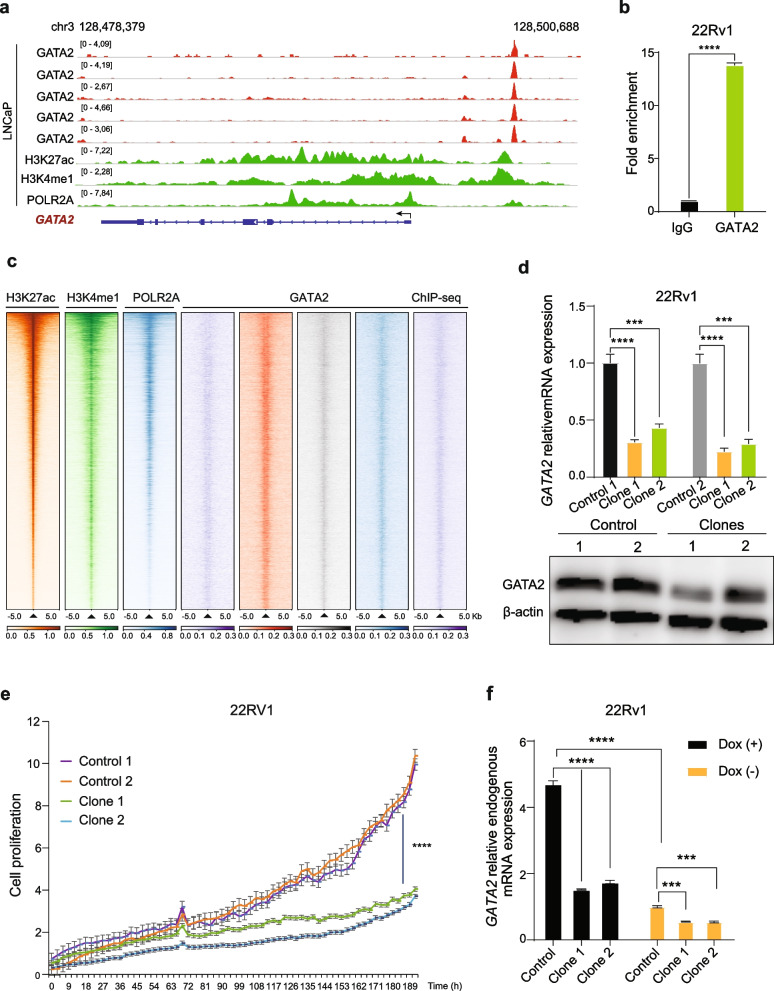
GATA2 drives its own expression via a positive feedback regulatory circuit. **a** Genome browser representation of ChIP-seq signals of active enhancer marks (H3K27ac and H3K4me1), RNA polymerase II (POLR2A), and transcription factor GATA2 at 3q21.3 *GATA2* locus in the LNCaP cells. **b** ChIP-qPCR analysis of GATA2 chromatin binding at the GATA2 enhancer in 22Rv1 cells. **c** Heatmaps of H3K27ac, H3K4me1, RNA polymerase II and GATA2 ChIP-seq signals around H3K27ac binding sites in LNCaP cells. H3K27ac binding sites were rank-ordered based on H3K27ac ChIP-seq intensities. **d** CRISPR/Cas9-mediated deletion of the GATA2-occupied GATA2 enhancer. GATA2 expression was analyzed by qRT-PCR and western blot in the two positive clones and control cells, β-actin used as loading control. **e** Cell growth potential was measured in real time by the Incucyte detecting system. The cell proliferation rate was detected every three hours. **f** Ectopic expression of GATA2 in the enhancer knockout clones showed a lower mRNA expression of endogenous GATA2 comparing to control cells

Having established that GATA2 amplification is positively correlated with its elevated expression in PCa patient tumors and the expression levels of GATA2 are markedly upregulated over tumor progression to advanced stage and metastasis, displaying profound associations with poor clinical outcomes in PCa, we next sought to elucidate the mechanism underlying aberrant transcription control of GATA2 overexpression. We therefore searched Cistrome DB with over 20,000 publicly available human genome-wide ChIP-seq datasets [64] and found that the transcription factor GATA2 itself indicates most regulatory potential over its genomic region in PCa cells (Fig. S2a). Indeed, we observed strong binding sites of GATA2 in its transcriptional start region (Fig. 2a). Furthermore, we performed ChIP-qPCR on the GATA2 locus demonstrating a strong enrichment of GATA2 in this region (Fig. 2b), thus providing compelling evidence for autoregulation.

Notably, GATA2 binding signals were co-enriched in the genomic regions with H3K27ac and H3K4me1, the epigenetic hallmarks of active typical enhancers or super-enhancers, as well as the occupancy of RNA polymerase II (POLR2A; Fig. 2c) [65, 66], indicating that GATA2 is profoundly involved in global gene transcription programs. Consistent with this notion, GATA2 has been proven to frequenctly and densely occupy within super-enhancers and promotes a robust gene transcription program to maintain mast cell identity [67]. To determine whether GATA2 binds to strong enhancers to augment its own expression, we next ranked GATA2-bound active enhancers per gene by binding signal, and found that the super-enhancer in the GATA2 locus was a top ranked GATA2 target in PCa cells (Fig. S2b). To examine if the GATA2 enhancer directly regulates GATA2 expression, we conducted functionally CRISPR/Cas9-mediated deletion of the GATA2-bound enhancer region. By qRT-PCR and Western blot assays, we found that deletion of the enhancer region reduced GATA2 mRNA and protein expression in PCa cells 22Rv1 (Fig. 2d). In line with declined expression of GATA2, using the Incucyte detecting system, we observed decreased cell proliferation in the enhancer-deleted clones compared to the parental cells (Fig. 2e). To further examine whether the GATA2-bound enhancer is responsible for GATA2 expression, we performed ectopic expression of GATA2 in the enhancer knockout clones and control cells, respectively. Using qRT-PCR with primers specific to endogenous GATA2 (see Materials and Methods), we found that while ectopically forced expression of GATA2, the mRNA levels of endogenous GATA2 increased by 1.52 and 1.72 folds in clone 1 and 2 cells, respectively, which were markedly lower than the control cells with increased expression of 4.71 folds (Fig. 2f), further supporting that the GATA2-bound enhancer is mediating its own expression.

We next explored the possibility of other transcription factor occupancies at this region in regulating GATA2 expression, and thus conducted an analysis via the Cistrome ToolKit [68]. The result suggested that the transcription factors including GATA2, CREB1, E2F1, and MYC may bind to the enhancer region in LNCaP cells (Fig. S2c). To further examine whether the predicted transcription factors could regulate GATA2 expression, we retrieved and analyzed RNA-seq profiling data upon perturbation of CREB1, E2F1, or MYC as previous studies reported in LNCaP cells [58, 69, 70]. The results of differential gene expression analysis indicated that knockdown of CREB1 or E2F1 showed no impact on GATA2 expression (Fig. S2d, e), while overexpressing MYC slightly downregulated GATA2 expression with a low-evidential *P* value (fold change = 0.69, fdr = 0.017, Fig. S2f). We also performed E2F1 knockdown assay followed by qRT-PCR experiments to show that E2F1 indicates no impact on GATA2 expression (Fig. S2g). Overall, these results indicate that except for GATA2, these predicated transcription factors are less likely to regulate GATA2 expression through the above-studied GATA2 enhancer. Collectively, these data may demonstrate that the GATA2 super enhancer demarcated with H3K4me1 and H3K27ac promotes GATA2 expression and GATA2 binds to this region, thereby forming a positive feedback regulatory circuit to augment its own expression in PCa.

### GATA2 potentiates PCa cell proliferation and metastasis

**Fig. 3:**
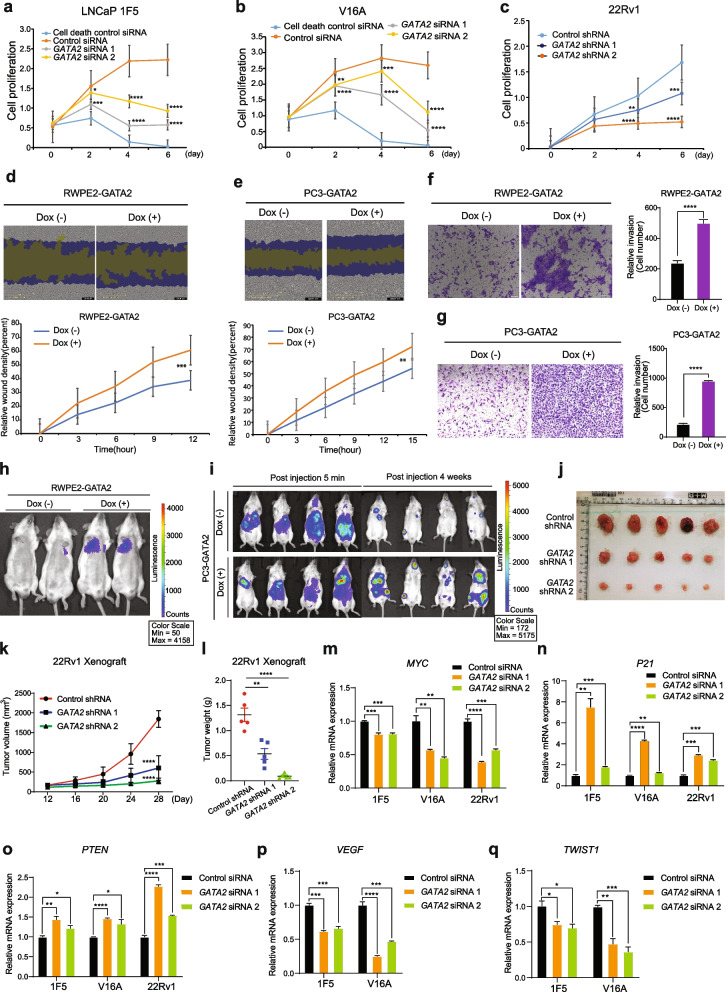
GATA2 potentiates PCa proliferation and metastasis. **a-c** Knockdown of GATA2 inhibited cell proliferation. Cells were transfected with control or GATA2 siRNAs or shRNAs, and cell proliferation was measured by XTT assay (absorbance at 450 nm) at the indicated time points. **d-e** Dox-induced GATA2 overexpression in RWPE2 (**d**) and PC3 cells (**e**) potentiated cell migration determined by wound healing assay. **f**-**g** GATA2 promoted cell invasion in RWPE2 (**f**) and PC3 (**g**) cells. **h-i** GATA2 promoted PCa metastasis in vivo. SCID male mice were tail-vain injected with RWPE2-GATA2 cells (**h**) or left ventricle injected with PC3-GATA2 cells (**i**), then administrated water with or without Dox (2 μg/mL). After 4 weeks, the lung (**h**) or bone (**i**) metastatic nodules and fluorescence intensity were measured by IVIS. **j**-**l** GATA2 promoted prostate cancer cell proliferation in vivo. SCID male mice were subcatenously inoculated with 22Rv1 scramble cells or GATA2 downregulated cells. Tumor size (**j**), tumor volume (**k**) and tumor weight (**l**) were measured. **m-q** GATA2 knockdown repressed cell cycle progression driver *MYC* (**m**) and promoted the expression of cell proliferation inhibitor *P21* (**n**) as well as stimulated the expression of metastasis suppressor *PTEN* (**o**) and inhibited metastasis drivers *VEGF* (**p**) and *TWIST1* (**q**) examined by qRT-PCR. Error bars are mean s.e.m, n = 3 independent experiments. ^*^P < 0.05, ^**^P < 0.01, ^***^P < 0.001, ^****^P < 0.0001 determined by unpaired Student’s *t*-test

To further investigate the role of GATA2 upregulation in PCa, we performed siRNA or shRNA-mediated knockdown of GATA2 in diverse PCa cell lines, including LNCaP, LNCaP-1F5 (1F5), V16A and 22Rv1 (Fig. S3a, b). As shown in Fig. 3a-c, cells with siRNAs or shRNAs against GATA2 significantly attenuated cell proliferation when comparing to the cells transfected with control siRNA or shRNA, consistent with genome-wide CRISPR/Cas9-screening data [14] showing GATA2 as the most essential gene for PCa cell survival (Fig. 1a) and previous report, indicating that depletion of GATA2 reduced cell proliferation and migration of LNCaP cells [71]. In addition, we introduced GATA2 into a Dox-inducible gene expression vector and established a series of GATA2 overexpression stable cell lines in RWPE2, 1F5, V16A, 22Rv1 and PC3 cells, namely RWPE2-GATA2, 1F5-GATA2, V16A-GATA2, 22Rv1-GATA2 and PC3-GATA2, respectively. We observed phenotypic difference under microscope between RWPE2-GATA2 and parental RWPE2 cells. As shown in Fig. S3c, the parental epithelial RWPE2 cells shared a round morphology, whereas RWPE2-GATA2 cells showed an irregular elongated and spindle shape, which is the typical morphology of mesenchymal cells [72], indicating a carcinogenic neoplastic transformation. Further, we analyzed cell migration property in these stable cell lines by performing wound-healing assay. The results showed an apparent wound closure in Dox-inducible group compared to vehicle (control group) (Fig. 3d, e and Fig. S3d-f). Moreover, we performed Transwell cell migration and invasion assays. RWPE2, PC3 and V16A cells with GATA2 overexpression showed increased cell migration (Fig. S3g-i). Similarly, GATA2 upregulation increased cell invasive capability of RWPE2, PC3, 1F5, 22Rv1 and V16A cells, respectively (Fig. 3f,g and Fig. S3j-l). In light of the robust clinical correlation between GATA2 upregulation and PCa metastasis as described above (Fig. 1e, h, j-m, p and Fig. S1g), we next experimentally substantiated the role of GATA2 in metastasis and inoculated RWPE2-GATA2 cells or PC3-GATA2 cells into SCID male mice via the tail vein injection or left ventricle injection. Using an in vivo imaging system (IVIS) upon 4 weeks post-injection, we observed Dox-inducible group mice showing more lung or bone metastasis compared to control group (Fig. 3h, i), demonstrating that overexpression of GATA2 promoted cell metastasis in vivo. To further verify the role of GATA2 in tumor cell proliferation in vivo, we subcutaneously inoculated the shRNA-mediated GATA2 knockdown 22Rv1 cells or cells with shRNA-scramble into SCID male mice by right flank injection. As shown in Fig. 3j-l, the tumor volume and weight were lower in GATA2 knockdown groups in comparision to control groups, thereby suggesting that GATA2 promotes prostate cancer cell proliferation in vivo. Lastly, we examined the expression of cell proliferation and metastasis related genes in GATA2 knockdown cell lines, 1F5, V16A and 22Rv1 by qRT-PCR analysis. The results showed that depletion of GATA2 downregulated the mRNA levels of cell proliferation driver gene *MYC* [73] while upregulated the cell growth inhibitor genes *P21* [74] and *PTEN* [75] (Fig. 3m-o). Moreover, downregulation of GATA2 attenuated the expression of cell metastasis relevant genes *VEGF* [76] and *TWIST1* [77] (Fig. 3p, q). Collectively, these results established the importance of GATA2 for PCa cell proliferation and metastasis, further strengthening the abovementioned clinical links between GATA2 alterations including amplification and upregulation and PCa tumor aggressive phenotype.

### GATA2 physically interacts and is cooperative with SMAD4 for genome-wide chromatin co-occupancy and co-regulation of PCa genes and cancer metastasis pathways

**Fig. 4:**
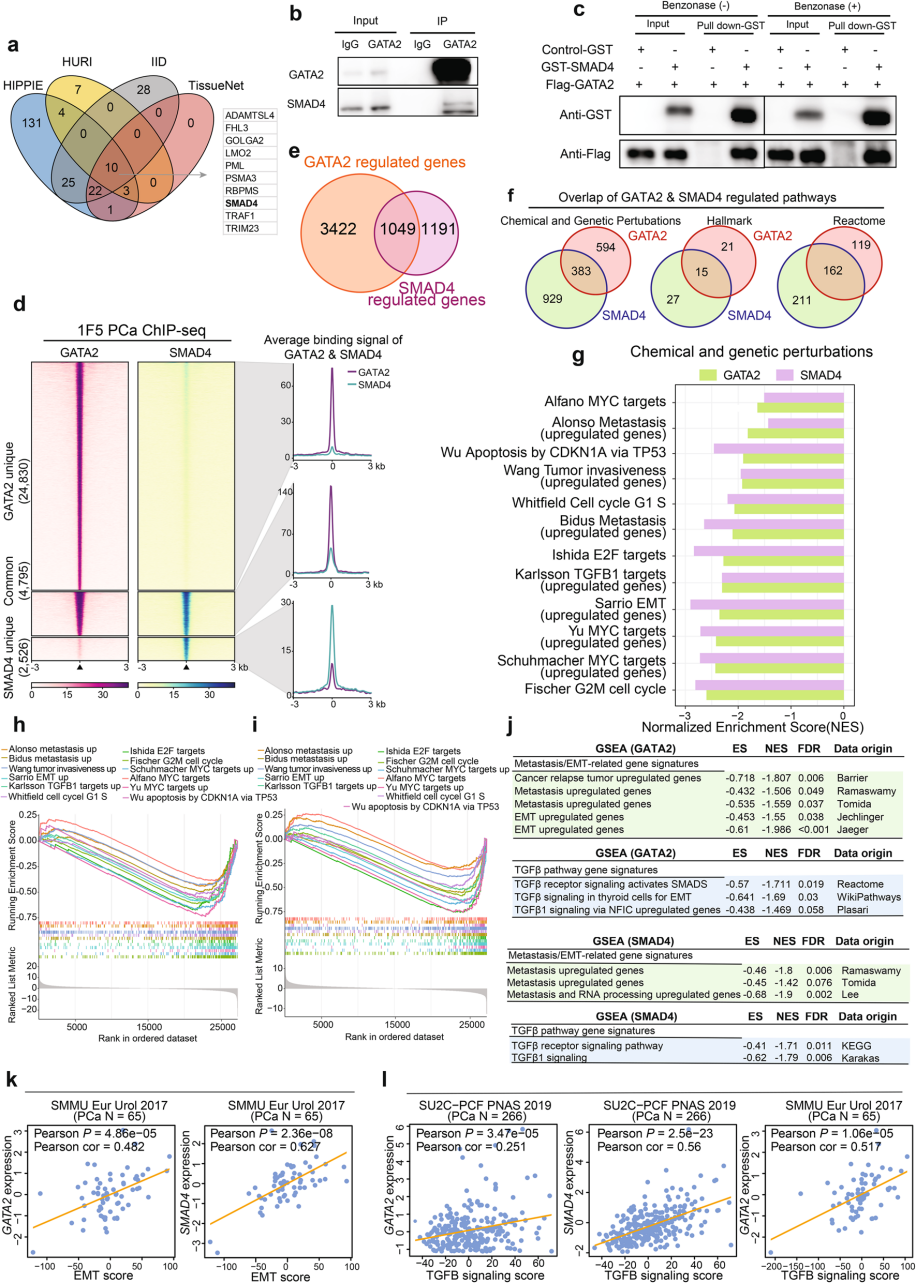
GATA2 physically interacts with and co-opts to SMAD4 for genome-wide chromatin co-occupancy and co-regulation of PCa genes and cancer metastasis pathways. **a** Proteins interacting with GATA2 in four indenpdent PPI databases. 10 proteins were condordently identified to interact with GATA2 in the four databases. **b** Interaction between endogenous GATA2 and SMAD4 was examined by immunoprecipitation (IP) using LNCaP cell lysates. **c** Directly interaction between GATA2 and SMAD4 was confirmed by GST-pull down assay. **d** Heatmap representation of GATA2 and SMAD4 chromatin binding intensities within 3 kb around the center of binding peaks in 1F5 cells. ChIP-seq signals were displayed in a descending order for clustered categories of GATA2 unique, GATA2 and SMAD4 common, and SMAD4 unique binding regions. **e** Venn diagram exhibiting common differentially expressed genes upon knockdown of GATA2 or SMAD4 followed by RNA-seq in 1F5 cells. FDR < 0.1. **f** Common regulated pathways of GATA2 and SMAD4 from MSigDB gene sets Chemical and Genetic Perturbations, Hallmark and Reactome. FDR < 0.05. **g** Several functional categories including cell cycle progression, metastasis and TGFβ signaling commonly enriched with downregulated genes upon siRNA-mediated knockdown of GATA2 or SMAD4 in 1F5 cells. **h-i** GSEA plots displaying pathways related to cell cycle progression, metastasis and TGFβ signaling enriched in GATA2 (**h**) or SMAD4 (**i**) upregulated genes. **j** Validation of common enriched pathways, which include metastasis/EMT-related gene and TGFβ pathway gene signatures, from GATA2 and SMAD4 target gene sets in additional resources. **k-l** Expression levels of GATA2 or SMAD4 were significantly correlated with the EMT score (**k**) or TGFβ signaling score (**l**) in PCa tumors

As a pioneer transcription factor, GATA2 has capability of binding to the DNA regions of closed chromatin, initiating hierarchical recruitment and occupancy of other regulatory proteins and forming a complicated transcriptional protein complex, which provide a promising therapeutic strategy by disrupting the protein–protein interactions (PPIs) in PCa treatment [15, 23]. To better understand oncogenic functions of GATA2 overexpression and amplification, we conducted a comprehensive query to identify GATA2-interacting proteins in four independent PPI databases [78–81]. To strengthen GATA2 interactors with high confidence we intersected the four obtained PPI lists. This analysis revealed 10 proteins including SMAD4 as the most confident protein that may directly interacts with GATA2 (Fig. 4a). To investigate the importance of these GATA2-interactors on cell survival, we mapped the 10 genes to the genome-wide CRISPR/Cas9-based loss-of-function screen data [14]. The result showed that SMAD4, as the only transcription factor among the 10 proteins identifed as potential GATA2 interactors, was fundamentally top-ranked as an essential gene for PCa cell survival (Fig. S4a), indicating that the potential interaction between GATA2 and SMAD4 is likely to be functionally important for PCa cell growth and tumor progression. To further confirm these findings, we constructed an expression vector of Flag-GATA2 for analyzing its interaction with SMAD family members (SMADs) in 293T cells. The result showed that SMAD4 indicated the strongest binding with GATA2 (Fig. S4b). To consolidate the results, both Flag-GATA2 and V5-SMAD4 were ectopically expressed in 293T cells and immunoprecipitated with Flag antibody, SMAD4 was apparently detected in the precipitates (Fig. S4c). Reciprocally, GATA2 can also be examined in the precipitates when immunoprecipitated with V5 antibody (Fig. S4d). This is in line with a previous report of in vitro interaction of GATA2 with SMAD4 in 293T cells [82]. To further prove if they interact in vivo in PCa cells, we performed endogenous co-IP experiments and found indeed bona fide interaction between GATA2 and SMAD4 in cultured LNCaP cells (Fig. 4b). To test whether GATA2 directly interacts with SMAD4, we conducted GST pull down assay and confirmed a direct interaction between GATA2 and SMAD4 in 1F5 cells (Fig. 4c). We next sought to rule out the possibility of DNA or RNA in mediating the interaction, and thus applied Benzonase to digest the DNA and RNA before pull down experiments. Compared to the untreated group, we still observed constant direct interaction between GATA2 and SMAD4 under Benzonase treatment (Fig. 4c), thus further strengthening the direct physical interaction between GATA2 and SMAD4. Strikingly, in line with their physical interaction, ChIP-seq analysis of GATA2 and SMAD4 genome-wide binding sites showed that over 65% (4795/7321) of SMAD4 chromatin-associated regions were co-occupied by GATA2 in 1F5 cells (Fig. 4d and Fig. S4e).

To reveal the mechanisms underlying GATA2 and SMAD4 cooperation and function in PCa, we conducted transcriptome-wide RNA sequencing (RNA-seq) in 1F5 cells upon siRNA-mediated knockdown of GATA2 and SMAD4, respectively. Two biological replicates were included in each group and high correlations were determined between the replicates (Fig. S4f-j). The RNA-seq revealed that 2,109 and 2,362, or 1,186 and 1,054 genes were significantly up- and downregulated, respectively, upon GATA2 or SMAD4 knockdown in 1F5 cells (Fig. S4k, l), suggesting that GATA2 and SMAD4 profoundly influence gene expression programs in PCa. Given that GATA2 and SMAD4 physically interact and co-bind a large propotion of genomic regions, we reasoned that GATA2 and SMAD4 may contribute to PCa progression via co-regulating genes converged on shared category of functional pathways. To prove this, we first examined the amount of common dysregulated genes revealed by RNA-seq profiling and observed that strikingly, near 50% of SMAD4 targeting genes were jointly co-regulated by GATA2 (Fig. 4e). We next performed GSEA [53] using the Molecular Signature Database (MSigDB) [54], and identified over 40% of common enriched pathways between GATA2 and SMAD4 in the collection of Chemical and Genetic Perturbations, Hallmark and Reactome database (Fig. 4f). Intriguingly, among these co-regulated functional categories, GATA2 and SMAD4 jointly modulated the expression of genes involved in multiple cancer-promoting pathways particularly like TGFβ signaling that is required for tumor invasiveness and metastasis (Fig. 4g). GSEA enrichment plots further indicated that these functional pathways reinforcing cell proliferation, epithelial-mesenchymal transition (EMT) and metastasis were highly enriched in upregulated genes targeted by GATA2 or SMAD4 in PCa cells (Fig. 4h, i). To further consolidate these findings, we carried out additional enrichment analysis in a large collection of annotated gene sets, the results coherently revealed the strong enrichment of genes downregulated upon GATA2 or SMAD4 knockdown for pathways relevant with cell proliferation (Fig. S4m), cancer metastasis and TGFβ signaling (Fig. 4j). To assess if these findings hold clinical impacts, we further performed correlations of these pathways with the expression of GATA2 or SMAD4 in several independent cohorts of PCa patient tumors, and found that GATA2 or SMAD4 expression levels were significantly and positively correlated with EMT scores (Fig. 4k and Fig. S4n) and TGFβ singaling scores (Fig. 4l and Fig. S4o), respectively. Taken together, our results and analyses demonstrate that GATA2 and SMAD4 display physical protein interaction in vitro and in vivo, and are commonly associated with genome-wide chromatin binding and transcriptome-wide PCa gene expression, thereby regulating common oncogenic pathways known to drive cancer cell proliferation and metastasis.

### GATA2 interacts and cooperates with SMAD4 to promote TGFβ1 signaling

**Fig. 5:**
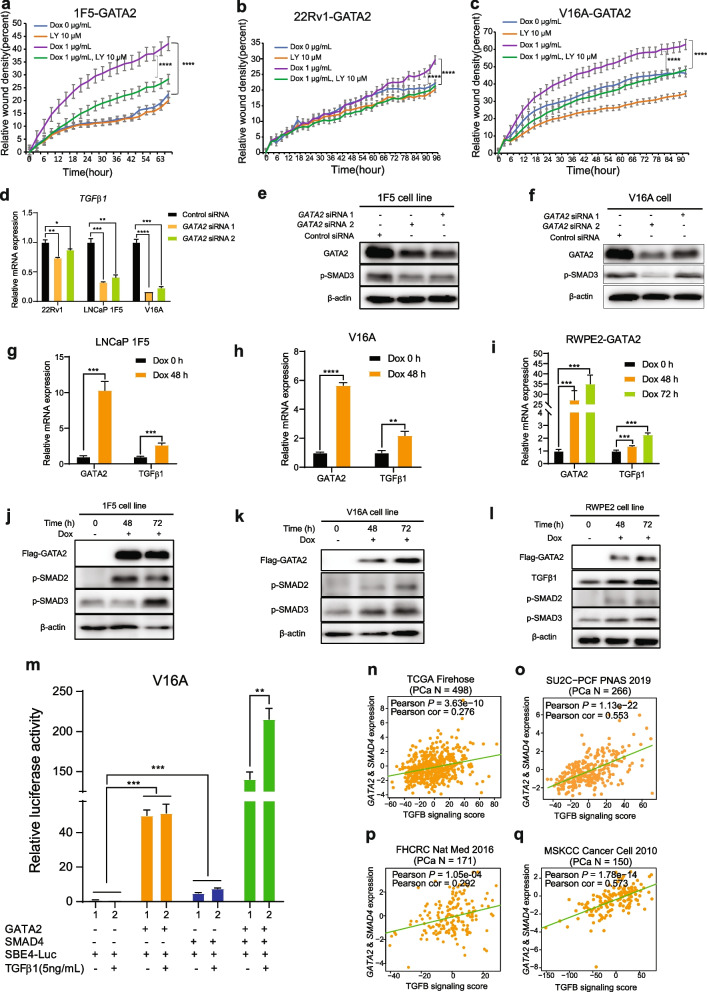
GATA2 cooperates with SMAD4 to promote TGFβ1 signaling. **a-c** TGFβ signaling inhibitor LY2157299 compromised GATA2-induced cell migration. 1F5-GATA2 (**a**), 22Rv1-GATA2 (**b**) and V16A-GATA2 (**c**) cells were treated with or without Dox (1 μg/ml) and LY2157299 (10 μM). Cell migration was determined by wound healing assay. **d** Knockdown of GATA2 decreased the expression of TGFβ1. 1F5, V16A and 22Rv1 cells were transfected with control siRNA or siRNAs specifically targeted on GATA2. Seventy-two hours later, the mRNA expression level of TGFβ1 was analyzed by qRT-PCR. **e–f** Downregulation of GATA2 suppressed the activity of TGFβ1/SMAD signaling. 1F5 and V16A cells were transfected with control siRNA or siRNAs against GATA2. Seventy-two hours later, the protein expression levels of GATA2 and p-SMAD3 were analyzed by western blot, β-actin used as loading control. **g-l** Upregulation of GATA2 activated TGFβ1/SMAD signaling. Cells were incubated with or without Dox (1 μg/ml) for 48 h or 72 h. Then the cell pellets were collected and the expression of TGFβ1, p-SMAD2 and p-SMAD3 were detected by qRT-PCR and western blot, respectively. **m** V16A cells were co-transfected with SBE4-luc, GATA2 or SMAD4 with or without 5 ng/mL TGFβ1 recombinant protein. Forty-eight hours later, cell luciferase activity was measured. **n-q** Significant positive linear expression correlation between GATA2 & SMAD4 and TGFβ signaling scores was observed in multiple independent PCa cohorts. *P* values assessed by the Pearson's product-moment correlation test. n = 3 independent experiments. ^**^P < 0.01, ^***^P < 0.001, ^****^P < 0.0001 determined by unpaired Student’s *t*-test

Given that TGFβ signaling pathway is commonly enriched in GATA2 and SMAD4 regulated genes, we examined whether TGFβ signaling involves in GATA2-mediated migration in PCa cells. Thus, we treated 1F5-GATA2, 22Rv1-GATA2 and V16A-GATA2 cells with TGFβ pathway inhibitor LY2157299 (LY) [83] and monitored real-time cell migration via wound healing assays at the indicated time points. As shown in the Fig. 5a-c, overexpression GATA2 promoted PCa cell migration, while treated with LY, the relative wound density caused by GATA2 was compromised, indicating that TGFβ signaling is indeed involved in GATA2-promoted cell migration.

To gain insight into the regulation of key TGFβ pathway genes that might be directly regulated by GATA2, we performed qRT-PCR. The results showed that downregulation of GATA2 specifically and consistently attenuated the mRNA expressions of *TGFβ1* (Fig. 5d), but not other TGFβ signaling ligand and receptor genes (Fig. S5a-e). It is well established that increased levels of p-SMAD2/3 mark the activation of TGFβ1 signaling pathway [84]. Consistent with the finding of GATA2’s function in positively regulating TGFβ1 expression (Fig. 5d), Western blotting demonstrated that knockdown of GATA2 caused an apparently decreased p-SMAD3 level (Fig. 5e, f) while GATA2 overexpression markedly promoted the expression of TGFβ1 and p-SMAD2/3 levels (Fig. 5g-l and Fig. S5f, g). Furthermore, to test whether GATA2 can drive TGFβ-SMAD signaling responsiveness, we utilized a luciferase reporter harboring four copies of the SMAD binding site (SBE4) [85] that are known to be activated by SMAD4. Unexpectedly, we found that GATA2 outperformed SMAD4 to strongly induce reporter activity and the SBE4-driven luciferase activity dramatically enhanced when co-expressed GATA2 and SMAD4 (Fig. 5m). To consolidate the result, we treated cells with the TGFβ1 recombinant protein. As shown in Fig. 5m, TGFβ1 further enhanced GATA2- and SMAD4-directed reporter activity but showed little influence on the GATA2 activation to SBE4, suggesting that GATA2 interacts and synergize with SMAD4 to promote the TGFβ1 signaling in PCa. To assess clinical impact of these findings, we computed TGFβ signaling scores across multiple independent PCa cohorts and showed that the expression levels of GATA2 and SMAD4 greatly correlated with TGFβ signaling activity in human PCa tumors (Fig. 5n-q and Fig. S5h), further strengthening that GATA2 is cooperative with SMAD4 to potentiate TGFβ1 signaling in PCa cells possibly in the clinical settings.

### GATA2 directly binds to a distal enhancer of TGFβ1 and regulates TGFβ1 expression in PCa cells

**Fig. 6:**
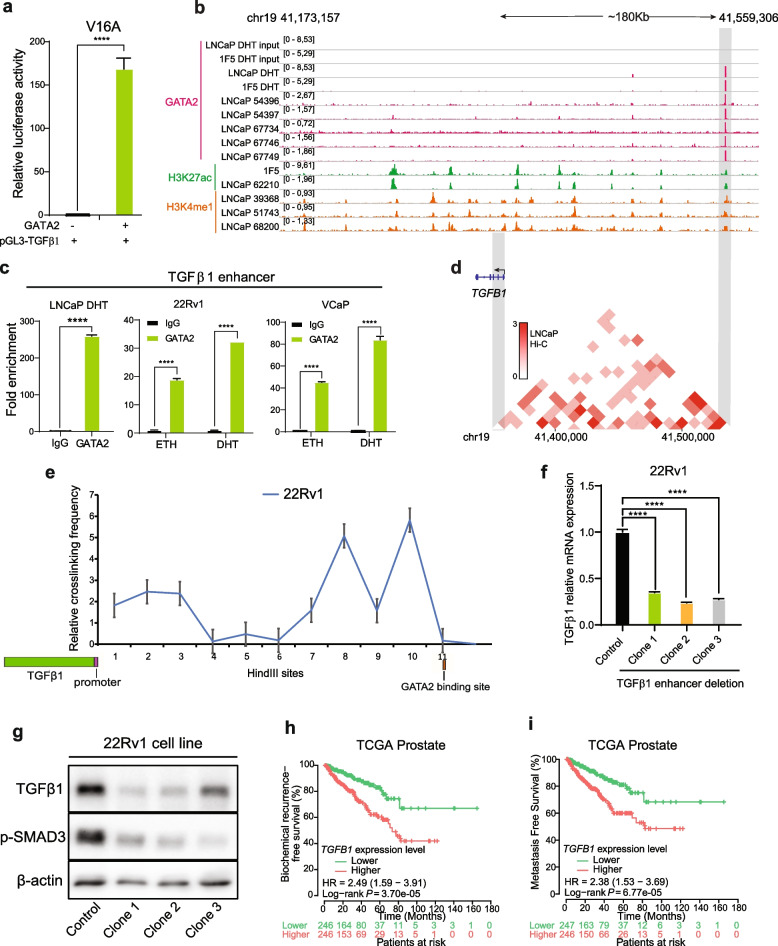
GATA2 binds to a distal enhancer of TGFβ1 and regulates TGFβ1 expression in PCa cells. **a** Luciferase reporter assays showing increased promoter activity of TGFβ1 when co-transfected with GATA2 expression vector in V16A cells. **b** Genome browser representations of GATA2 ChIP-seq enriched profiles at far upstream of TGFβ1 in PCa LNCaP and 1F5 cells. Chromosome coordinates presented the human genome build hg38. **c** ChIP-qPCR verification of GATA2 chromatin binding at the TGFβ1 upstream enhancer region in LNCaP, 22Rv1 and VCaP cells. **d** Hi-C analysis of chromatin interactions between the potential GATA2-occupied TGFβ1 enhancer and TGFβ1 promoter locus (chr19: 41,355,000–41,531,050). **e** 3C-qPCR analysis of chromatin interactions between the enhancer locus and TGFβ1 promoter region (chr19: 41,355,000–41,531,050). **f-g** CRISPR/Cas9-mediated deletion of the GATA2-occupied TGFβ1 enhancer. 22Rv1 cells were transfected with control or TGFβ1 enhancer-targeting sgRNAs. Three clones were picked up and confirmed by Sanger sequencing. TGFβ1 expression was analyzed by qRT-PCR (**f**) and western blot (**g**) in the three clones and control cells, β-actin used as loading control. **h-i** Kaplan Meier plots indicated increased biochemical recurrence and metastasis risks of PCa patients with higher TGFβ1 expression levels in TCGA cohort. *P* values assessed by log-rank test. All the error bars are mean s.e.m, *n* = 3 independent experiments. ^****^P < 0.0001, determined by unpaired Student’s *t*-test

Since GATA2 positively regulating the expression of TGFβ1 (Fig. 5d, g-h), we next asked whether *TGFβ1* is a direct target gene of GATA2. To test the hypothesis, we first performed a promoter luciferase reporter assay in V16A cells and observed that GATA2 profoundly stimulated the activity of TGFβ1 promoter (Fig. 6a). We next reanalyzed GATA2 ChIP-seq data across different PCa cell models and noticed a strong GATA2 binding site locating at approximately 180 kb upstream of TGFβ1 gene promoter (Fig. 6b). We performed ChIP-qPCR to confirm GATA2 binding to this site in the PCa cell lines LNCaP, 22Rv1 and VCaP, respectively (Fig. 6c). To explore whether there is direct chromatin interactions between this GATA2 binding site and TGFβ1 promoter region, we first queried the Hi-C data of PCa cells LNCaP from the 3D genome browser [86] and observed a likely remote interaction between these two regions (Fig. 6d). We further conducted quantitative chromosome conformation capture assays (3C-qPCR) [87] using the restriction enzyme Hind III. TGFβ1 promoter region was set up as constant fragment, and its interactions were assessed with Hind III-digested chromatin fragments in this 200 kb genomic window. The results showed that this GATA2 binding site has higher crosslinking frequencies in PCa cells 22Rv1 (Fig. 6e).

Having established that a distal GATA2 bind site upstream of TGFβ1 can form chromatin interaction with TGFβ1 promote region in PCa cells, we next investigated whether the putative enhancer directly regulates TGFβ1 expression. We thus applied functional CRISPR/Cas9 editing system using two independent pairs of single guide RNA (sgRNA) sequences flanking the GATA2 binding sites to delete the region in PCa cells 22Rv1 (Table S6). We picked up three independent clones with successful deletion of GATA2 binding sites after a series of experimental confirmations (see Materials and Methods; Fig. S6a, b). Deletion of the GATA2 binding sites greatly reduced TGFβ1 mRNA and protein expression as well as the abundance of p-SMAD3 in PCa cells, indicating attenuated TGFβ signaling upon knockout of this GATA2 binding site (Fig. 6f, g). Finally, by performing a Kaplan–Meier survival analysis, we found that human PCa patients with tumors expressing higher levels of TGFβ1 showed apparently increased probability of biochemical relapse and metastasis (Fig. 6h, i). Taken together, we provide supporting evidence that GATA2 binds to an upstream enhancer of TGFβ1 to promote its expression, which in turn may activate TGFβ1 signaling pathway contributing to PCa progression.

### GATA2 co-opts with SMAD4 to regulate AR signaling and PCa risk genes

**Fig. 7:**
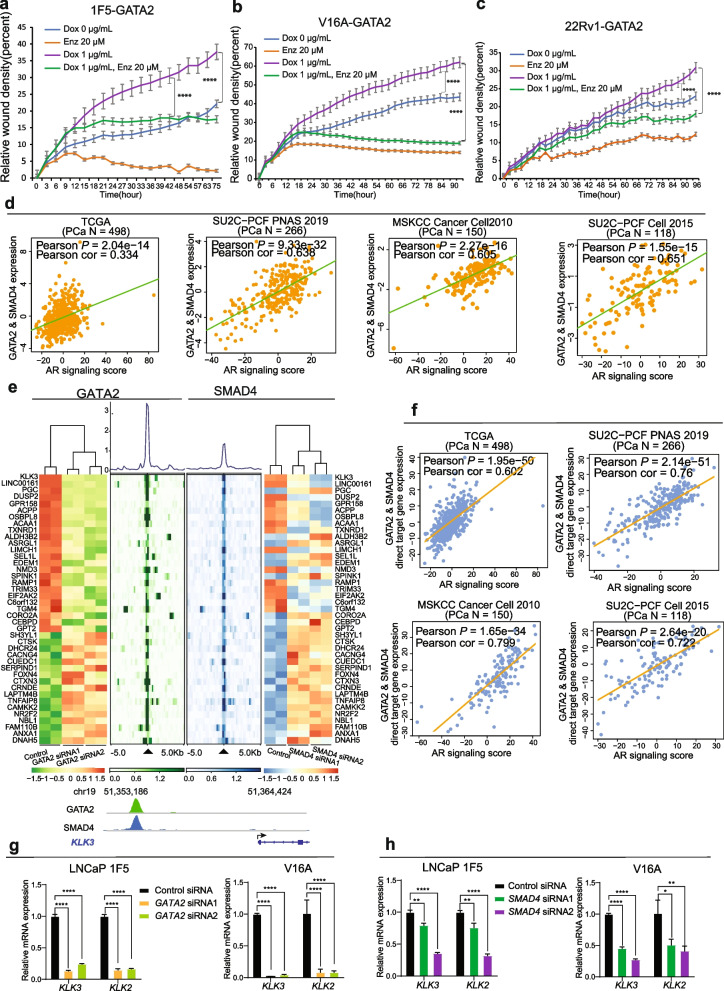
GATA2 co-opts with SMAD4 to regulate AR signaling. **a-c** AR signaling antagonists Enzalutamide compromised GATA2-driven cell migration in 1F5 (**a**), V16A (**b**) and 22Rv1 (**c**) cells. **d** GATA2 and SMAD4 expression levels positively correlated with AR signaling scores in human PCa tumors in multiple independent cohorts. *P* values examined by the Pearson's product-moment correlation test. **e** Upper panel: Integrated heatmaps of RNA-seq and ChIP-seq representations for the joint direct targeting genes of GATA2 and SMAD4 identified in 1F5 cells. GATA2 and SMAD4 ChIP-seq signals were illustrated for the genes shown, deeper color indicating higher enrichment. Lower panel: chromatin-binding of GATA2 and SMAD4 on the proxy region of representative PCa gene *KLK3* with indicated genomic interval. **f** The Z-score sum of expression levels of GATA2 & SMAD4 direct target gene signature showed positive linear correlation with AR signaling score in human PCa tumors in multiple independent cohorts. *P* values examined by the Pearson's product-moment correlation test. **g-h** Knockdown of GATA2 (**g**) or SMAD4 (**h**) inhibited the mRNA expression levels of AR targeting genes *KLK2* and *KLK3* in 1F5 and V16A cells. All the error bars represent s.e.m, *n* = 3 technical replicates. ^*^P < 0.05, ^**^P < 0.01, ^****^P < 0.0001, determined by unpaired Student’s *t*-test

It was previously reported that GATA2 is critical for AR expression and proper transcriptional activity [20]. Thus, we reasoned that AR signaling might participate in regulating GATA2-induced PCa cell invasiveness. To test this, we treated 1F5, V16A and 22Rv1 cells individually with Enzalutamide (Enz), an AR antagonists. Indeed, our results from real time monitoring wound healing assays confirmed that Enz significantly compromised GATA2 overexpression-driven cell migration (Fig. 7a-c).

Given that GATA2 and SMAD4 share many commonly regulated pathway categories implicated in PCa progression, we next explored the clinical significance of their association with AR signaling status and observed that the joint expression levels of GATA2 and SMAD4 strikingly correlated with AR signaling scores across multiple independent clinical PCa datasets (Fig. 7d and Fig. S7a, b). We next sought to explore whether GATA2 and SMAD4 joint direct target genes show associations with AR signaling in PCa. We thus performed an integrated analysis of RNA-seq transcriptome profilings upon GATA2 or SMAD4 knockdown and their genome-wide chromatin occupancy data in 1F5 PCa cells (Fig. S4k, l), thereby resulting in 41 direct target genes of GATA2 and SMAD4 (Fig. 7e, upper panel). Notably, a well-known AR targeting gene *KLK3*, coding prostate-specific antigen (PSA) was top ranked as a most significantly downregulated gene upon GATA2 knockdown in 1F5 cells, with strong chromatin binding of GATA2 and SMAD4 at the upstream enhancer of *KLK3* (Fig. 7e, lower panel and Fig. S7c). Consistently, *KLK3* downregulation was also detected in SMAD4 knockdown followed by RNA-seq profiling (Fig. S7d). Given that GATA2 and SMAD4 positively correlated with AR signaling in PCa patient tumors (Fig. 7d and Fig. S7a, b), we next examined whether the GATA2 and SMAD4 direct target gene signature correlates with AR signaling. Indeed, this analysis revealed strikingly positive linear correlations in multiple independent PCa cohorts (Fig. 7f and Fig. S7e, f).

PCa is a type of most heritable cancer and genome-wide association studies (GWAS) have discovered PCa risk- or aggressiveness-associated non-coding variants, often regulating gene expression (eQTL) through modulating transcription factor-DNA binding [40, 41, 88, 89]. Therefore, we asked whether GATA2 and SMAD4 possess genetic impact on PCa risk associations. We subsequently incorporated GWAS identified PCa risk single nucleotide polymorphism (SNP) loci and retrieved their proxy SNPs in tight linkage disequilibrium (LD, R^2^ ≥ 0.5), and computed the enrichment of these SNP-containing regions in the GATA2 or SMAD4 ChIP-seq peaks across multiple PCa cell lines (see Materials and Methods). We next determined eQTL genes (eGenes) that are associated to the SNPs enriched in GATA2 or SMAD4 binding sites from three resources of eQTL datasets, including GTEx [90], PancanQTL [91] and ncRNA-eQTL [92], and revealed a dozen of eGenes influenced by GATA2 or SMAD4 (Fig. S7g, h; see Materials and Methods). Strikingly, *KLK3* was identified as an eGene with both GATA2 and SMAD4 chromatin occupancy spanning its eQTL SNP-containing region (Fig. 7e), thereby defined as both a direct target gene and an eGene based on integrated multilayers of genetic and genomic data in PCa. Our qRT-PCR assays further validated that knockdown of GATA2 or SMAD4 reduced the mRNA expression levels of AR signaling targeting genes, *KLK2* and *KLK3* in 1F5 and V16A cells, respectively (Fig. 7g, h. Collectively, these data suggest that GATA2 co-opts with SMAD4 to regulate the AR signaling in PCa and genetically defined PCa risk- or aggressiveness-associated genes.

### GATA2 and SMAD4 show a global impact on PCa risk-associations and forms a transcriptional complex with HOXB13 to drive the expression of PCa risk gene RFX6 at 6q22

**Fig. 8:**
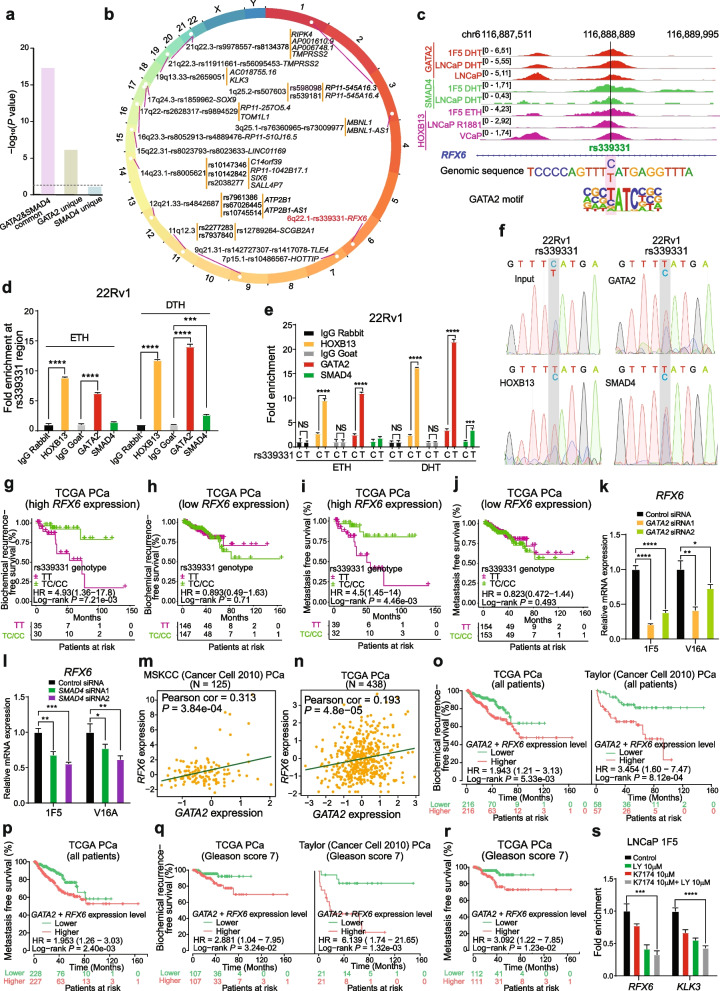
GATA2 shows a global impact on inherited PCa risk and forms a transcriptional complex with SMAD4 and HOXB13 at rs339331/6q22 enhancer to drive the expression of PCa risk gene *RFX6*. **a** The enrichment analysis indicated a substantial increase for PCa GWAS risk SNPs enriched in the common chromatin binding sites of GATA2 and SMAD4. **b** Circos overview of PCa risk loci enriched in the GATA2 and SMAD4 common chromatin binding regions in 1F5 cells. The outer ring represented a circular ideograph of the human genome annotated with chromosome numbers. Tag SNPs were positioned in each locus followed by corresponding proxy SNPs with a cutoff LD, R^2^ ≥ 0.5. The eQTL genes were indicated adjacent to proxy SNPs. **c** Genome browser represented of rs339331 residing within GATA2, SMAD4 and HOXB13 ChIP-seq chromatin binding regions in PCa cell lines. Lower panel: rs339331 is located within the GATA2 DNA-binding motif. Chromosome coordinates indicate as the human genome build hg38. **d** ChIP-qPCR for GATA2 and SMAD4 chromatin binding at the rs339331-containing region in 22Rv1 cells after ETH or DHT (100 nM) treatment for 24 h. HOXB13 was shown as as a positive control. **e**-**f** GATA2 and SMAD4 favor binding to the T risk allele than C at rs339331 as determined by ChIP followed by AS-qPCR (**e**) and ChIP followed by PCR amplification and Sanger sequencing (**f**). **g-j** PCa tumors carrying rs339331 risk allele TT were associated with shorter biochemical recurrence-free (**g**) and metastasis-free (**i**) survival in patient group expressing higher RFX6 levels. rs339331 could not stratifiy PCa patient with lower RFX6 expression (**h**, **j**). *P* values assessed by the log-rank test. **k-l** Knockdown of GATA2 or SMAD4 decreased the expression of *RFX6* in LNCaP 1F5 and V16A cells. **m**–**n** Scatter plot illustration of linear correlation between GATA2 and RFX6 expression levels in human prostates. **o**-**p** Kaplan–Meier plots demonstrated increased risks of biochemical recurrence and metastasis for PCa patients with elevated GATA2 and RFX6 expression levels in TCGA and Taylor cohorts. **q-r** Synergistic expression effect of RFX6 and GATA2 exhibited predictive values for biochemical relapse and metastasis in PCa patient group with an intermediate risk of Gleason Score 7. Samples with RFX6 deep loss were ruled out in the analysis. Patients were stratified by the median value of RFX6 and GATA2 expression levels. *P* values assessed by the log-rank test. **s** The effect of combination TGFβ signaling inhibitor and GATA2 inhibitor on the expression of *RFX6* and *KLK3* in 1F5 cells. LNCaP cells were treated with or without 10 μM K7174 together with 10 μM LY2157299 for 48 h. qRT-PCR was following performed to analyze the mRNA expression of the correlated genes. All the error bars represent s.e.m, *n* = 3 technical replicates. ^*^P < 0.01 ^**^P < 0.01, ^***^P < 0.001, ^****^P < 0.0001, determined by two-tailed Student’s *t*-test. N.S: Non-significant

We next investigated whether GATA2 and SMAD4 together synergistically explain more impacts on genetic predisposition to PCa. We thus calculated the enrichment of PCa risk SNPs in the binding regions of GATA2 alone, GATA2 and SMAD4 common as well as SMAD4 alone, respectively. Intriguingly, we found that PCa risk SNPs were greatly enriched in the common binding sites of GATA2 and SMAD4 in comparison to their individual counterparts (Fig. 8a). We then extracted enriched risk SNPs and integrated multi-sources of eQTL data to define a detailed locus-SNP-eGenes association Circos map (Fig. 8b), pinpointing a potential functional association among GATA2, SMAD4 and the PCa susceptibility locus at 6q22 harboring the GWAS-reported variant rs339331 that was mechanistically investigated in our previous study [40]. We previously demonstrated that the transcription factor HOXB13 preferably binds to the risk-associated T allele at rs339331, leading to increased expression of RFX6 and potential contribution to PCa pathogenesis [40]. Herein, the observed chromatin bindings of GATA2 and SMAD4 at rs339331-containing region (Fig. 8c) motivated us to explore whether the variants at rs339331 might modulate a more sophisticated transcription factor complex including not only HOXB13 but also GATA2 and SMAD4, thereby additively altering RFX6 expression.

We next conducted bioinformatic prediction to examine a direct impact of variation at rs339331 on transcription factor DNA-binding motifs, and revealed that GATA2 favor the binding to the PCa risk-associated T allele of rs339331 (Fig. 8c and Fig. S8a). To verify these findings independently, we first performed ChIP-qPCR and confirmed obvious enrichment of GATA2 and SMAD4 at the rs339331-containg region both in PCa cells 22Rv1 (Fig. 8d) and VCaP (Fig. S8b). As a positive control, HOXB13 is well-established to bind at this chromatin region [40, 93]. Using 22Rv1 and VCaP cell models that are heterozygous for rs339331, we next performed allele specific (AS) ChIP-qPCR and revealed that GATA2, SMAD4 and HOXB13 all were preferentially binding to T allele than C allele of rs339331 (Fig. 8e and Fig. S8c). Consistent with this, Sanger sequencing results showed also higher profiles of the rs339331 T-allele-containing region for the ChIPed DNA of GATA2, SMAD4 and HOXB13, respectively than that of input (Fig. 8f). We next examined whether rs339331 genotype could directly correlate with PCa patient survival. The results indicated that rs339331 genotypes was not associated with PCa patient prognosis (Fig. S8d, e). Given that the risk allele at rs339331 was associated with higher expression of RFX6 and that RFX6 upregulation correlated with PCa progression [40], we thus explored whether rs339331 together with RFX6 expression status may synergistically impact PCa prognosis. We therefore stratified PCa patients into two groups with tumors expressing RFX6-high or -low levels and examined the direct link of rs339331 to PCa patient prognosis in each group. Intriguingly, we found that the PCa patients with tumors having higher expression levels of RFX6 while carrying homozygous risk genotype TT at rs339331 were strongly associated with increased risk of biochemical recurrence and metastasis (Fig. 8g, i). In contrast, we found no association in PCa patients with tumors displaying lower RFX6 expression (Fig. 8h, j). Therefore, these results show that the PCa patients carrying rs339331 risk allele TT with tumors expressing higher levels of the eQTL gene RFX6 are associated with poor prognosis in PCa.

Our previous study [94] reported a novel gene regulatory mechanism underlying the risk SNP loci in altering ternary transcription factor complexes, yet how the regulatory proteins hierarchically formed at a SNP-containing region remains illusive. To test this, we further investigated whether GATA2, SMAD4 and HOXB13 can mutually influence their binding at the rs339331-containing region, and thus established shRNA-mediated knockdown stable cell lines for each factor. Using standard AS ChIP-qPCR, we showed that knockdown of HOXB13 caused decreased binding of GATA2 but not SMAD4 at the rs339331 or its risk T allele-containing region (Fig. S8f, g), and vice versa, and GATA2 knockdown alleviated HOXB13 chromatin occupancy but not SMAD4 at this region (Fig. S8h, i). When knocking down SMAD4, chromatin binding of GATA2 but not HOXB13 to the rs339331 region was greatly altered (Fig. S8j, k), indicating that SMAD4 locates in the upstream of GATA2 and HOXB13 at the rs339331-enhancer-mediated formation of transcription factor complex. Consistent with these chromatin binding data, our qRT-PCR results showed that siRNA-mediated downregulation of GATA2 or SMAD4 reduced the expression levels of *RFX6* (Fig. 8k, l), demonstrating that the rs339331 eGene *RFX6* is a direct target of GATA2 and SMAD4.

We next investigated the correlation between RFX6 and GATA2 expression in the clinical settings and observed a significant linear positive expression correlation between GATA2 and RFX6 in multiple independent PCa datasets (Fig. 8m, n and Fig. S9a). Based on our findings as described above, GATA2 exerted prognostic value in predicting PCa patient survival (Fig. 1g, h, o-q) and our previous study showing that RFX6 possesses clinical impact on PCa progression [40], we hence asked whether RFX6 together with GATA2 synergistically perform better in clinical PCa prognosis. We thus examined the synergistic effect of RFX6 and GATA2 in multiple cohorts of PCa patients [5]. The Kaplan–Meier survival analysis displayed that PCa patients with tumors expressing higher levels of RFX6 and GATA2 were associated with increased risk for biochemical relapse and metastasis (Fig. 8o, p). Intriguingly, RFX6 and GATA2 together demonstrated higher hazard ratios compared to that of RFX6 or GATA2 alone in PCa risk prediction (Fig. S9b-d). We next examined whether the expression levels of RFX6 together with GATA2 exert better predictive values in patient group with intermediate risk (Gleason score 7) compared to their individual counterparts. We thus subdivided PCa patients into three groups by Gleason score ≤ 6, 7 or ≥ 8. The survival analysis showed a predictive value for the joint expression levels of RFX6 and GATA2 for biochemical relapse and metastasis in the patients with Gleason score of 7, but not for the individuals with lower- (Gleason score 6) or higher risk (Gleason score 8) (Fig. 8q, r and Fig. S9e-g). Notably, we found that for patient group with Gleason score 7, compared to individuals having tumors expressing higher level of RFX6 or GATA2, patients with PCa tumors expressing both higher levels of RFX6 and GATA2 were at significantly higher risks of biochemical relapse and metastasis (Fig. S9h-m). These findings indicat that RFX6 and GATA2 together display a superior synergistic predictive value for PCa in the clinical settings.

Having established that the TGFβ/SMAD pathway inhibitor LY compromises GATA2-induced PCa cell growth and invasiveness (Fig. 5a-c), together with a previous study showing that GATA2 inhibitor K7174 blocks the recruitment of GATA2 to transcriptional target genes [22], we sought to examine whether GATA2 and SMAD4-regulated eQTL genes can be potential therapeutic targets for PCa. We thus treated LNCaP 1F5 cells with LY, K7174, or their combination (Fig. 8s). qRT-PCR analysis showed that combined GATA2 inhibitor K7174 and TGFβ/SMAD signaling inhibitor LY indeed decreased the expression levels of the 6q22 *RFX6* and 19q13.33 *KLK3* genes (Fig. 8s). We next found a similar synergistic effect of K7174 and LY on other eQTL genes, including *TMPRSS2*, *SIX-6, ATP2B1-AS1*, *TLE4*, *HOTTIP* and *MBNL1* (Fig. S9n, o), suggesting a potential clinical application with the identified eQTL genes, their upstream signaling pathways and transcription factors in PCa.

## Discussion

**Fig. 9:**
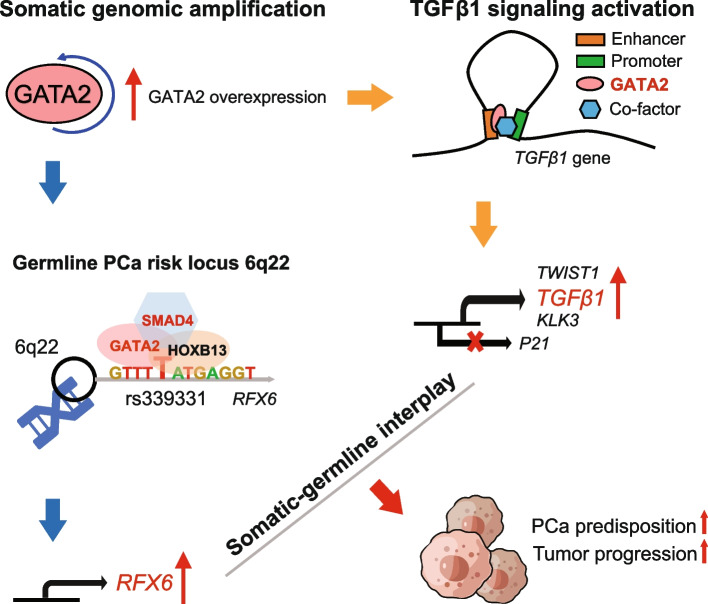
An extensive mechanistic cooperation between GATA2 and TGFβ1/SMAD4 signaling contributes to PCa predisposition and progression. Schematic showing that prevalent genomic copy gain of GATA2 in PCa and a previously unappreciated autoregulation mechanism direct GATA2 overexpression, thereby promoting PCa cell proliferation and metastatic progression. Mechanistically, GATA2 cooperates with SMAD4 physically and on chromatin, and drives the expression of TGFβ1 via a distal enhancer, hence activating TGFβ1/SMAD4 signaling and orchestrating decreased expression of cell cycle inhibitor *P21* as well as enhanced transcription of metastasis-associated genes, such as *TGFβ1* and *TWIST1*. Moreover, GATA2 is cooperative with SMAD4 and the prostate-lineage-specific transcription factor HOXB13 to mediate inherited PCa risk, indicating chromatin-binding preference to the 6q22 PCa risk-asociated T allele of the SNP rs339331, resulting increased expression of the eGene RFX6 contributing to PCa severity. Collectively, GATA2 upregulation contributes to PCa predisposition and tumor progression through controlling oncogenic signaling and this extensive somatic-germline interplay mechanism in PCa

Emerging evidene implies the important role of GATA2 in PCa progression, indicating it as a potential target for the development of therapeutic strategies [15]. In this study, we found that GATA2 is top-ranking among the highly amplified genes in cancer genomes of PCa patients and indicates as the most essential gene for PCa cell survival, showing positive correlations with its elevated expression in tumors of PCa patient mostly accompanied with higher Gleason score, advanced tumor stage, elevated PSA levels and shorter biochemical recurrence-free survival time, consistent with previous studies also showed that GATA2 is upregualted in PCa and its upregulationcorrelates with poor prognosis of PCa patients [20, 62, 63, 95]. We also found that GATA2 displays as an independent prognostic marker in distinguishing the intermediate-risk patients with PCa that may recur, further suggesting the potential value of GATA2 in PCa diagnosis and prognosis. Interestingly, we report for the first time the mechanism underlying GATA2 overexpression by which GATA2 binds to an upstream enhancer to drive its own expression via a positively autoregulated feedback loop in PCa (Fig. 9). Consistent with previous studies showing that downregulation of GATA2 inhibits PCa LNCaP cell proliferation and migration [71] and attenuates tumorigenicity in castration resistant prostate cancer [95], our data further showed that GATA2 overexpression potentiate PCa cell proliferation and metastasis both in vitro and in vivo, mechanistically, this is likely due to physical interaction between GATA2 and SMAD4 for genome-wide chromatin co-occupancy and co-regulation of PCa genes and metastasis pathways, including TGFβ and AR pathways.

TGFβ signaling pathway has been shown to significantly impact the cancer metastatic process [25, 26]. As a core component in TGFβ signaling, SMAD4 shows the highest response to TGFβ signaling by stabilizating or recruiting transcriptional factors and coactivators to gene regulatory elements in exquisite contexts [27, 28], leading to the pleiotropic roles of TGF-β/SMAD4 in cancer progression. A previous work reported that GATA2 inhibits TGFβ signaling via an interaction with SMAD4 in 293T cells [82]. Also, in murine hematopoietic progenitor cells, TGF-β/Smad4 and Gata2 forms a regulatory circuit to control the cell proliferation arrest gene p57 [96]. In this study, we showed that GATA2 physically interacted and was cooperative with SMAD4 for genome-wide chromatin co-occupancy in vivo and further enhanced TGFβ1 signaling to promote PCa metastasis. Mechanistically, we discovered that GATA2 directly bind to a distant enhancer region of TGFβ1 and increase its expression (Fig. 9). This mechanism can explain previous observations why overproduction of TGFβ1 is associated with angiogenesis, metastasis and poor clinical outcome in PCa [97]. Thus, GATA2 overexpression is likely to be a driving force for TGFβ1 upregulation and overall TGFβ pathway activation contributing to PCa progression.

Androgen signaling-dependent AR activation plays pivotal roles in both primary and metastatic PCa [98]. Previous studies have shown a global impact of GATA2 in transcriptional regulation of AR and AR targeting genes [17, 20–22]. Herein our data demonstrated a novel cooperation of GATA2 with SMAD4 to promote AR signaling. Given that previous studies have demonstrated the clinical utility of AR antagonists in both primary and metastatic PCa [99], especially Enzalutamide [100], our observation may provide a new insight into developing therapeutic strategy by inhibiting GATA2, TGFβ/SMAD4 and AR signaling together in PCa. Consistent with this hypothesis, recent studies have illustrated that combination of GATA2 inhibitor K7174 and AR antagonist enzalutamide suppress the proliferation of PCa cells [22] and combination of TGFβ signaling inhibitor LY2157299 and AR antagonist enzalutamide alleviate the proliferation and metastasis of PCa both in vitro and in vivo [101, 102], respectively. Thus, our results provide further insight to investigate the influence of combination of GATA2, TGFβ/SMAD4 and AR signaling inhibitors on the PCa progression.

PCa is a type of inheritable disease and genetic factor represents a main risk factor that contribute to PCa predisposition and proprogression. To discover the susceptibility loci for PCa, many GWAS projects have been initiated since 2005 and over 270 risk loci have been reported [35–37]. By investigating the molecular mechanisms underlying the biological effect of these risk SNPs, previous studies including ours suggest that these SNPs may affect gene regulation by modulating the binding of key transcription factors such as HOXB13, AR, and the most frequent PCa-specific fusion protein TMPRSS2-ERG [40, 41, 103] as well as the influence of genetic variants on transcription factor DNA-binding in other types of diseases [89, 104]. One of our pioneer studies demonstrates that the PCa risk-associated allele at rs339331 impacts PCa predisposition and progression by altering RFX6 expression through a functional interplay with the PCa susceptibility gene HOXB13 [40]. In this study, we expanded the work and further showed that GATA2 and SMAD4 together indicated a global impact on PCa risk associations, in particular forming a transcriptional complex with HOXB13 at rs339331 enhancer region to drive the expression of PCa risk gene RFX6 (Fig. 9). Moreover, here we demonstrated that the variants at rs339331 in PCa patients with tumors expressing high levels of *RFX6* possessed prognostic value on patient survival. The PCa patients carrying rs339331 risk allele TT and tumors with higher RFX6 expression were associated with increased chance of having biochemical recurrence and metastsatis. This synergisctic effect of rs339331 genotype and RFX6 expression might have clinical implications and translational value. We further showed that the combination of GATA2 and TGFβ signaling inhibitors efficiently attenuates the expression of PCa risk genes incluing *RFX6* and *KLK3*. Given that post-GWAS analysis help generate knowledge of gene networks and pathways, thereby further prioritizing therapeutic targets [105, 106], current study may serve as an example for exploring global impacts of GATA2 and SMAD4 on PCa risk associations and in particular expanding the underlying mechanisms on the diverse ancestry population associated PCa risk allele rs339331 to improve PCa risk prediction and develop therapeutic strategies against PCa.

## Conclusions

In summary, our data revealed that GATA2, a pioneer transcription factor with highly prevalent somatic genomic amplificaitons in PCa, interacts and cooperates with SMAD4 to promote PCa metastasis through activating TGFβ1 and AR signaling pathways. We also revealed two intricate transcriptional activation mechanisms by which GATA2 drives its own expression via transcriptional autoregulation and promotes TGFβ1 expression through directly binding to a distant enhancer of TGFβ1. Finally, we observed an extensive somatic-germline interplay among GATA2, SMAD4 and PCa risk loci, including the PCa risk-associated rs339331/RFX6 at 6q22 (Fig. 9). These findings may provide insights into further developing genetic marker for PCa prediction and therapy.

### Supplementary Information

**Additional file 1.**

**Additional file 2.**

## Data Availability

The RNA-seq, microarray or clinical data were obtained from the cBioPortal for Cancer Genomics [107, 108], Omcomine [109], GEO database [110] and literatures. HOXB13 and some of GATA2 ChIP-seq data were retrieved from Cistrome Data Browser (CistromeDB) [68]. The PCa GWAS associations were downloaded from the NHGRI-EBI GWAS Catalog [111]. The eQTL genes associated with enriched PCa SNPs were obtained from online eQTL datasets including GTEx portal [90], PancanQTL [91] and ncRNA-eQTL [92]. All public data used in this study were properly cited. Data generated or analyzed in this study are included in this article and the relevant supplementary files. The ChIP-seq and RNA-seq data generated in this study have been deposited to the ENA (European Nucleotide Archive) database under accession ID PRJEB62435 and RJEB62434, respectively.

## Abbreviations

PCa: Prostate cancer
co-IP: Coimmunoprecipitation
GWAS: Genome-wide association study
Dox: Doxycycline
ChIP: Chromatin immunoprecipitation
ChIP-seq: Chromatin immunoprecipitation sequencing
qRT-PCR: Quantitative Reverse Transcriptase - Polymerase Chain Reaction
RNA-seq: RNA sequencing
GSEA: Gene set enrichment analysis
AS-qPCR: Allele-specific quantitative RT-PCR
3C-qPCR: Quantitative analysis of chromosome conformation capture
PFA: Paraformaldehyde
Enz: Enzalutamide
GATA2: Endothelial transcription factor GATA-2
SMAD4: Sma Mothers Against Decapentaplegia homologue 4
SBE4: SMAD binding elements
DHT: Dihydrotestosterone
PSA: Prostate specific antigen
TGFβ: Transforming growth factor-β
CAD: Coronary artery disease
TSS: Transcription start site
