# Supplementary material for: GATA2 co-opts TGFβ1/SMAD4 oncogenic signaling and inherited variants at 6q22 to modulate prostate cancer progression
Source: J Exp Clin Cancer Res. 2023 Aug 8;42:198. doi: 10.1186/s13046-023-02745-7 (PMC10408074; doi:10.1186/s13046-023-02745-7)
Supplement: Supplementary file 1 — Additional file 1. [file 13046_2023_2745_MOESM1_ESM.pdf]

Fig. S1

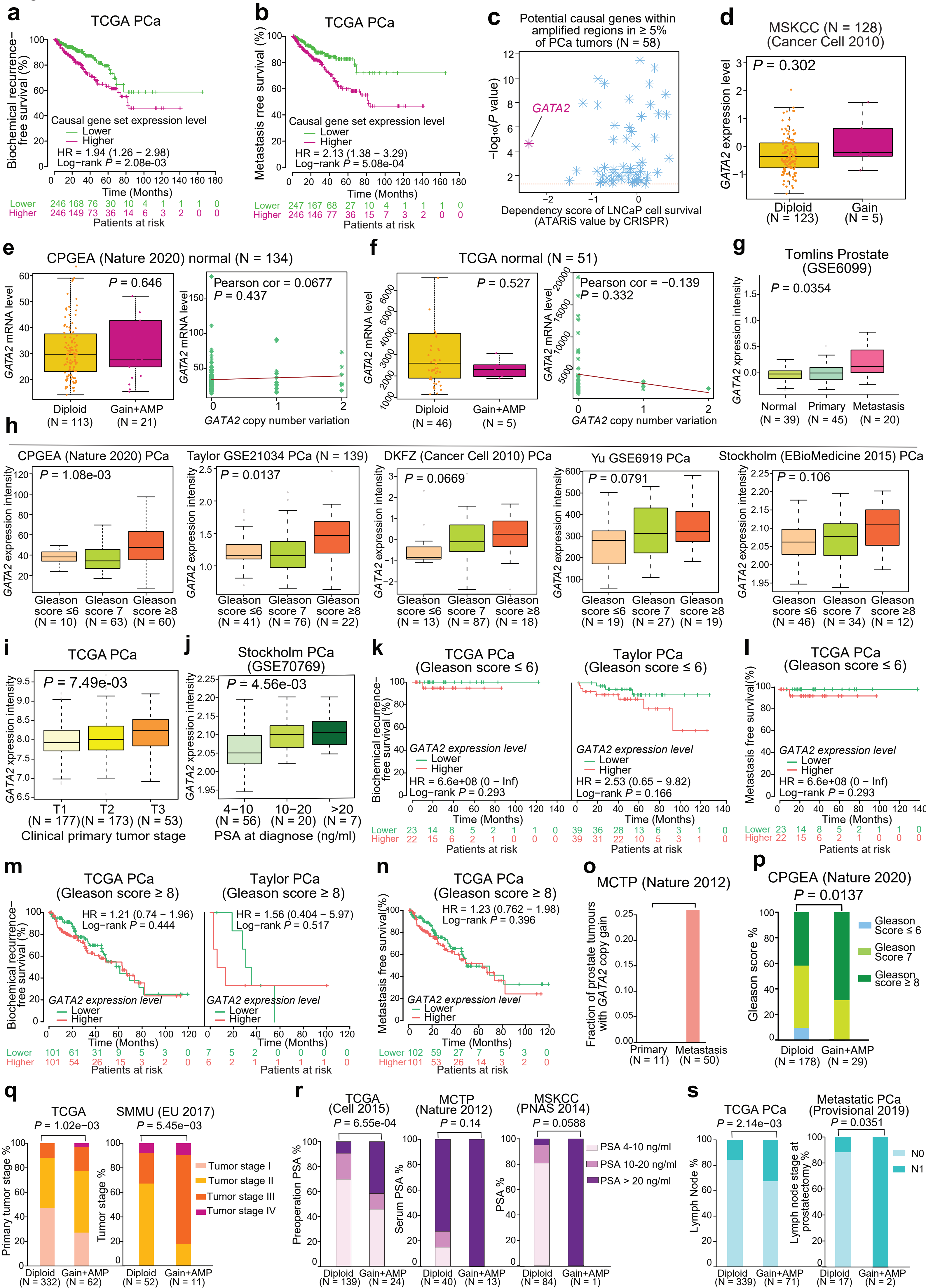
a
TCGA PCa
Biochemical recurrence-free survival (%)
Causal gene set expression level
Lower
Higher
HR = 1.94 (1.26 − 2.98)
Log−rank P = 2.08e-03
Time (Months)
Lower 246 168 76 30 10 4 1 1 1 0
Higher 246 149 73 36 14 6 3 2 0 0
Patients at risk
b
TCGA PCa
Metastasis rree survival (%)
Causal gene set expression level
Lower
Higher
HR = 2.13 (1.38 − 3.29)
Log−rank P = 5.08e-04
Time (Months)
Lower 247 167 68 27 10 4 1 1 0 0
Higher 246 146 77 36 15 7 3 2 0 0
Patients at risk
c
Potential causal genes within amplified regions in ≥ 5% of PCa tumors (N = 58)
−log10(P value)
GATA2
Dependency score of LNCaP cell survival (ATARiS value by CRISPR)
d
MSKCC (N = 128) (Cancer Cell 2010)
P = 0.302
GATA2 expression level
Diploid (N = 123)
Gain (N = 5)
e
CPGEA (Nature 2020) normal (N = 134)
P = 0.646
GATA2 mRNA level
Diploid (N = 113)
Gain+AMP (N = 21)
Pearson cor = 0.0677
P = 0.437
GATA2 copy number variation
f
TCGA normal (N = 51)
P = 0.527
GATA2 mRNA level
Diploid (N = 46)
Gain+AMP (N = 5)
Pearson cor = −0.139
P = 0.332
GATA2 copy number variation
g
Tomlins Prostate (GSE6099)
P = 0.0354
GATA2 xpression intensity
Normal (N = 39)
Primary (N = 45)
Metastasia (N = 20)
h
CPGEA (Nature 2020) PCa
P = 1.08e-03
GATA2 expression intensity
Gleason score ≤6 (N = 10)
Gleason score 7 (N = 63)
Gleason score ≥8 (N = 60)
Taylor GSE21034 PCa (N = 139)
P = 0.0137
Gleason score ≤6 (N = 41)
Gleason score 7 (N = 76)
Gleason score ≥8 (N = 22)
DKFZ (Cancer Cell 2010) PCa
P = 0.0669
Gleason score ≤6 (N = 13)
Gleason score 7 (N = 87)
Gleason score ≥8 (N = 18)
Yu GSE6919 PCa
P = 0.0791
Gleason score ≤6 (N = 19)
Gleason score 7 (N = 27)
Gleason score ≥8 (N = 19)
Stockholm (EBioMedicine 2015) PCa
P = 0.106
Gleason score ≤6 (N = 46)
Gleason score 7 (N = 34)
Gleason score ≥8 (N = 12)
i
TCGA PCa
P = 7.49e-03
GATA2 xpression intensity
T1 (N = 177)
T2 (N = 173)
T3 (N = 53)
Clinical primary tumor stage
j
Stockholm PCa (GSE70769)
P = 4.56e-03
GATA2 xpression intensity
4−10 (N = 56)
10−20 (N = 20)
>20 (N = 7)
PSA at diagnose (ng/ml)
k
TCGA PCa (Gleason score ≤ 6)
Biochemical recurrence-free survival (%)
GATA2 expression level
Lower
Higher
HR = 6.6e+08 (0 − Inf)
Log−rank P = 0.293
Taylor PCa (Gleason score ≤ 6)
GATA2 expression level
Lower
Higher
HR = 2.53 (0.65 − 9.82)
Log−rank P = 0.166
Time (Months)
Lower 23 14 8 5 2 1 1 0
Higher 22 15 6 2 1 0 0 0
Lower 39 36 28 13 6 3 1 0
Higher 39 31 22 10 5 3 1 0
Patients at risk
l
TCGA PCa (Gleason score ≤ 6)
Metastasis free survival(%)
GATA2 expression level
Lower
Higher
HR = 6.6e+08 (0 − Inf)
Log−rank P = 0.293
Time (Months)
Lower 23 14 8 5 2 1 1 0
Higher 22 15 6 2 1 0 0 0
Patients at risk
m
TCGA PCa (Gleason score ≥ 8)
Biochemical recurrence-free survival (%)
HR = 1.21 (0.74 − 1.96)
Log−rank P = 0.444
GATA2 expression level
Lower
Higher
Taylor PCa (Gleason score ≥ 8)
HR = 1.56 (0.404 − 5.97)
Log−rank P = 0.517
GATA2 expression level
Lower
Higher
Time (Months)
Lower 101 61 31 9 5 3 0
Higher 101 54 26 15 3 2 0
7 5 2 0 0 0 0
6 2 1 1 1 1 0
Patients at risk
n
TCGA PCa (Gleason score ≥ 8)
Metastasis free survival(%)
HR = 1.23 (0.762 − 1.98)
Log−rank P = 0.396
GATA2 expression level
Lower
Higher
Time (Months)
Lower 102 59 27 7 5 3 0
Higher 101 53 26 14 3 2 0
Patients at risk
o
MCTP (Nature 2012)
Fraction of prostate tumours with GATA2 copy gain
Primary (N = 11)
Metastasis (N = 50)
p
CPGEA (Nature 2020)
P = 0.0137
Gleason score %
Gleason Score ≤ 6
Gleason Score 7
Gleason score ≥ 8
Diploid (N = 178)
Gain+AMP (N = 29)
q
TCGA
P = 1.02e−03
Primary tumor stage %
Diploid (N = 332)
Gain+AMP (N = 62)
SMMU (EU 2017)
P = 5.45e−03
Tumor stage %
Diploid (N = 52)
Gain+AMP (N = 11)
Tumor stage I
Tumor stage II
Tumor stage III
Tumor stage IV
r
TCGA (Cell 2015)
P = 6.55e−04
Preoperation PSA %
Diploid (N = 139)
Gain+AMP (N = 24)
MCTP (Nature 2012)
P = 0.14
Serum PSA %
Diploid (N = 40)
Gain+AMP (N = 13)
MSKCC (PNAS 2014)
P = 0.0588
PSA %
Diploid (N = 84)
Gain+AMP (N = 1)
PSA 4-10 ng/ml
PSA 10-20 ng/ml
PSA > 20 ng/ml
s
TCGA PCa
P = 2.14e−03
Lymph Node %
Diploid (N = 339)
Gain+AMP (N = 71)
Metastatic PCa (Provisional 2019)
P = 0.0351
Lymph node stage at prostatectomy %
Diploid (N = 17)
Gain+AMP (N = 2)
N0
N1

Fig. S1 GATA2 amplification and upregulation are correlated with tumor progression and poor prognosis in PCa patients. a-b PCa patients showing higher expression levels of the amplified causal gene set were associated with shorter biochemical recurrence-free (**a**) and metastasis-free survival (**b**). **c** Volcano representation of causal genes with the genomic amplification rates over 5%. *P* values in y-axis were calculated with Mann–Whitney U test for examining the expression association with copy number gain levels. Lowest P values from the CPGEA or EU PCa cohort were represented. **d** GATA2 expression was higher in PCa patient group with GATA2 copy number gain. *P* value was assessed by the Mann–Whitney U test. **e-f** GATA2 mRNA level in normal prostate tissues with GATA2 wild type (diploid) or with matched tumors harboring copy number gain and gene amplification (left panel). Pearson correlation between GATA2 mRNA expression and copy number gain/amplification (right panel). Note that GATA2 copy number gain/amplification in the matched PCa tumors showed no apparent correlations with its upregulation in normal prostate samples. Gain: with presence of one copy; AMP, amplification: with the presence of two copies. *P* values determined by the Mann-Whitney U test or Pearson correlation. **g** Box plot showing GATA2 upregulation in human primary and metastasis PCa compared to normal prostate tissues. *P* values determined by the Kruskal-Wallis H test. **h** GATA2 was upregulated in PCa tumors with higher Gleason score. *P* values calculated by the Kruskal-Wallis H test. **i-j** Elevated GATA2 expression level correlated with higher tumor stage (**i**), and elevated PSA level (**j**). *P* values determined by the Kruskal-Wallis H test. **k-n** GATA2 expression levels cannot stratify PCa patients with lower (Gleason score ≤ 6, **k-l**) or higher risks (Gleason score ≥ 8, **m-n**) in multiple independent PCa cohorts. *P* values assessed by the log-rank test. **o** Fraction of tumors harboring GATA2 copy number gain/AMP was markedly higher in metastatic tumors compared to primary PCa. *P* values were examined by the Fisher's exact test. **p** Proportion of PCa patients with advanced Gleason scores was higher in group with GATA2 copy number gain/AMP in comparison with wild type (diploid). *P* values examined by the Fisher’s exact test. **q-s** PCa patients with GATA2 copy gain/AMP were markedly associated with advanced tumor stage (**q**), elevated PSA levels (**r**) and higher stage of lymph node metastasis (**s**). *P* values examined by Fisher’s exact test.

Fig. S2

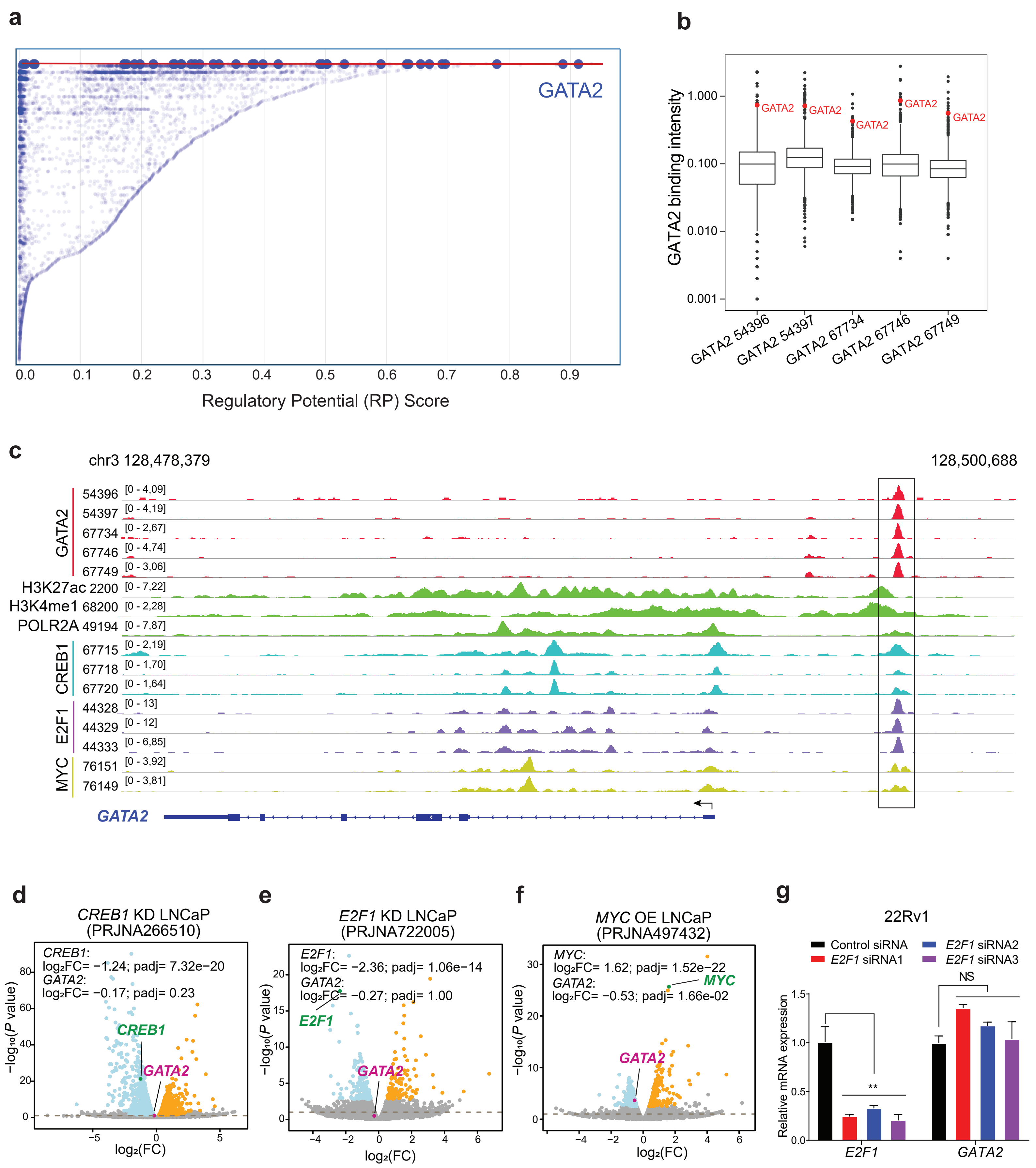

a
GATA2
Regulatory Potential (RP) Score
b
GATA2 binding intensity
GATA2 54396
GATA2 54397
GATA2 67734
GATA2 67746
GATA2 67749
c
chr3 128,478,379
128,500,688
GATA2
54396 [0 - 4,09]
54397 [0 - 4,19]
67734 [0 - 2,67]
67746 [0 - 4,74]
67749 [0 - 3,06]
H3K27ac 2200 [0 - 7,22]
H3K4me1 68200 [0 - 2,28]
POLR2A 49194 [0 - 7,87]
CREB1
67715 [0 - 2,19]
67718 [0 - 1,70]
67720 [0 - 1,64]
E2F1
44328 [0 - 13]
44329 [0 - 12]
44333 [0 - 6,85]
MYC
76151 [0 - 3,92]
76149 [0 - 3,81]
GATA2
d
CREB1 KD LNCaP (PRJNA266510)
CREB1: log₂FC= −1.24; padj= 7.32e−20
GATA2: log₂FC= −0.17; padj= 0.23
−log₁₀(P value)
log₂(FC)
e
E2F1 KD LNCaP (PRJNA722005)
E2F1: log₂FC= −2.36; padj= 1.06e−14
GATA2: log₂FC= −0.27; padj= 1.00
f
MYC OE LNCaP (PRJNA497432)
MYC: log₂FC= 1.62; padj= 1.52e−22
GATA2: log₂FC= −0.53; padj= 1.66e−02
g
22Rv1
Control siRNA
E2F1 siRNA1
E2F1 siRNA2
E2F1 siRNA3
NS
**
Relative mRNA expression
E2F1
GATA2

Fig. S2 GATA2 shows a most autoregulatory potential over its expression. a Regulatory potential of transcription factors regulating GATA2 expression. Big blue dots represented GATA2 transcription factor. X-axis represented the RP score while Y-axis indicated different transcription factors. Regulatory potential (RP) represented the estimate of how possible a transcription factor can regulate a gene. **b** Box plots depicting the distribution of GATA2 signal overactive enhancers defined by the overlap of H3K27ac, H3K4me1, and POLR2A in LNCaP cells. The active enhancer in the GATA2 locus was top ranked among GATA2 targets. Red dots represented GATA2 that ranked number 10, 20, 6, 12 and 11 in five illustrated GATA2 ChIP-seq profiles in LNCaP cells, respectively. **c** ChIP-seq tracks demonstrating the enrichment of active enhancer marks (H3K27ac and H3K4me1), RNA polymerase II (POLR2A), and transcription factors, including GATA2, CREB1, E2F1, and MYC at the 3q21.3 *GATA2* locus in the LNCaP cells. The CistromeDB ID of each ChIP-seq experiment were mentioned beside. The enhancer region was highlighted by the orange square. **d-f** RNA-seq analysis indicated the impact of knockdown of CREB1 (**d**) or E2F1 (**e**) or overexpression of MYC (**f**) to GATA2 expression in LNCaP cells. Differential gene expression was assessed by the DESeq2. **g** Downregulation of E2F1 showed little influence on GATA2 mRNA expression.

Fig. S3

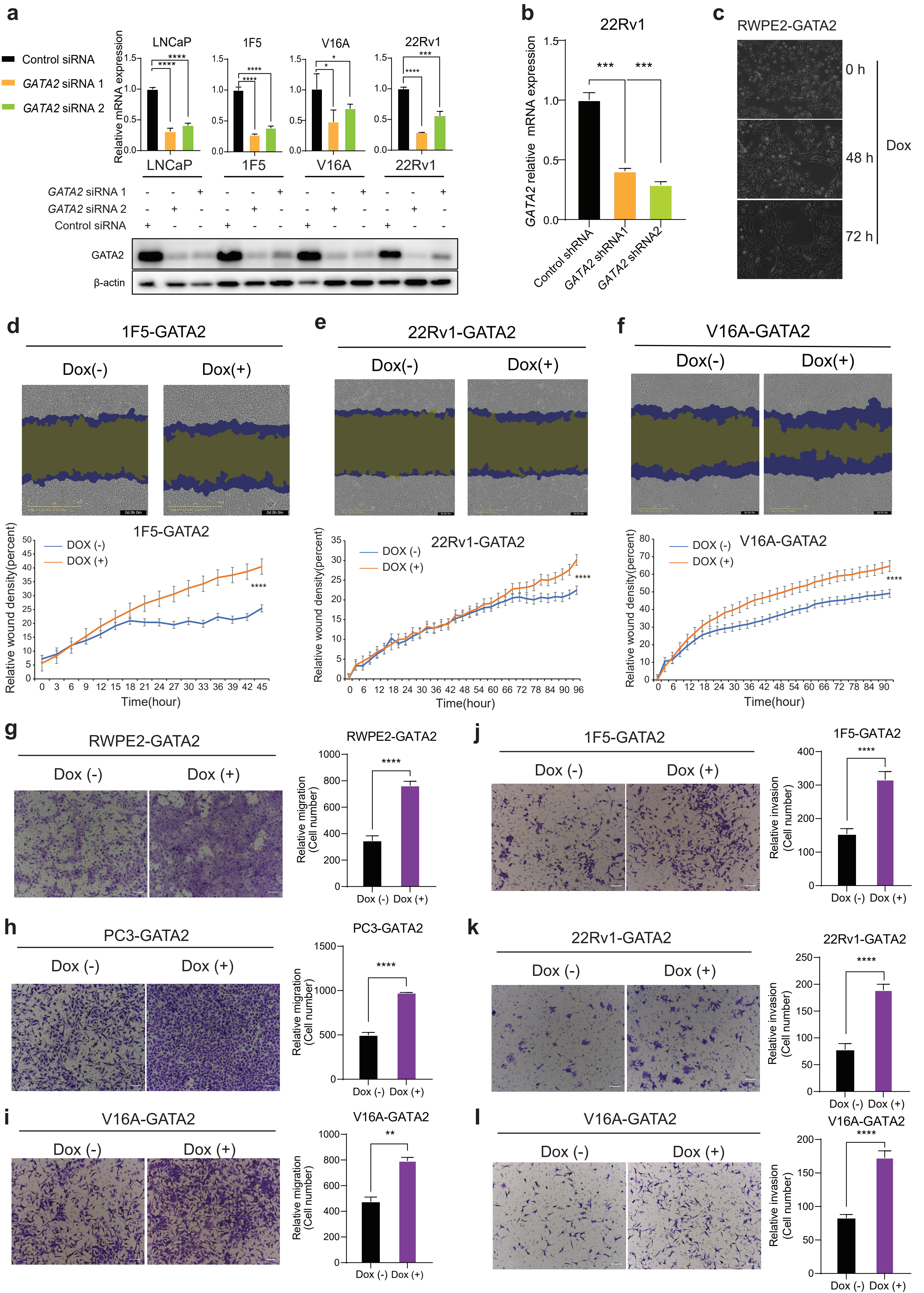
a
Control siRNA
GATA2 siRNA 1
GATA2 siRNA 2
Relative mRNA expression
LNCaP
1F5
V16A
22Rv1
GATA2 siRNA 1
GATA2 siRNA 2
Control siRNA
GATA2
β-actin
b
22Rv1
GATA2 relative mRNA expression
Control shRNA
GATA2 shRNA1
GATA2 shRNA2
c
RWPE2-GATA2
0 h
48 h
72 h
Dox
d
1F5-GATA2
Dox(-)
Dox(+)
DOX (-)
DOX (+)
Relative wound density(percent)
Time(hour)
e
22Rv1-GATA2
f
V16A-GATA2
g
RWPE2-GATA2
Dox (-)
Dox (+)
Relative migration (Cell number)
h
PC3-GATA2
i
V16A-GATA2
j
1F5-GATA2
Relative invasion (Cell number)
k
22Rv1-GATA2
l
V16A-GATA2

Fig. S3 GATA2 potentiates PCa proliferation and metastasis. a Knockdown efficiency of GATA2 by siRNAs in different PCa cells was measured by qRT-PCR and western blot. **b** Knockdown efficiency of GATA2 by shRNAs in 22Rv1 cells was measured by qRT-PCR. **c** Cell morphology was observed under optical microscope (20x) in RWPE2-GATA2 cells with or without Dox (1 μg/mL) in indicated time. **d-f** Overexpression GATA2 facilitated PCa cell migration. 1F5-GATA2, 22Rv1-GATA2 and V16A-GATA2 cells were treated with 1 μg/mL Dox and then wound density was measured at indicated time points in 1F5 (**d**), 22Rv1 (**e**) and V16A cells (**f**). **g-i** Overexpression of GATA2 promoted cell Transwell migration in RWPE2-GATA2 (**g**), PC3-GATA2 (**h**) and V16A-GATA2 (**i**) cells. **j-l** Overexpression of GATA2 potentiated cell invasion in 1F5-GATA2 (**j**), 22Rv1-GATA2 (**k**) and V16A-GATA2 (**l**) cells. All error bars represent s.e.m, n=3 independent experiments. $^{**}P<0.05$, $^{***}P<0.001$, $^{****}P<0.0001$, determined by unpaired Student's *t*-test.

Fig. S4

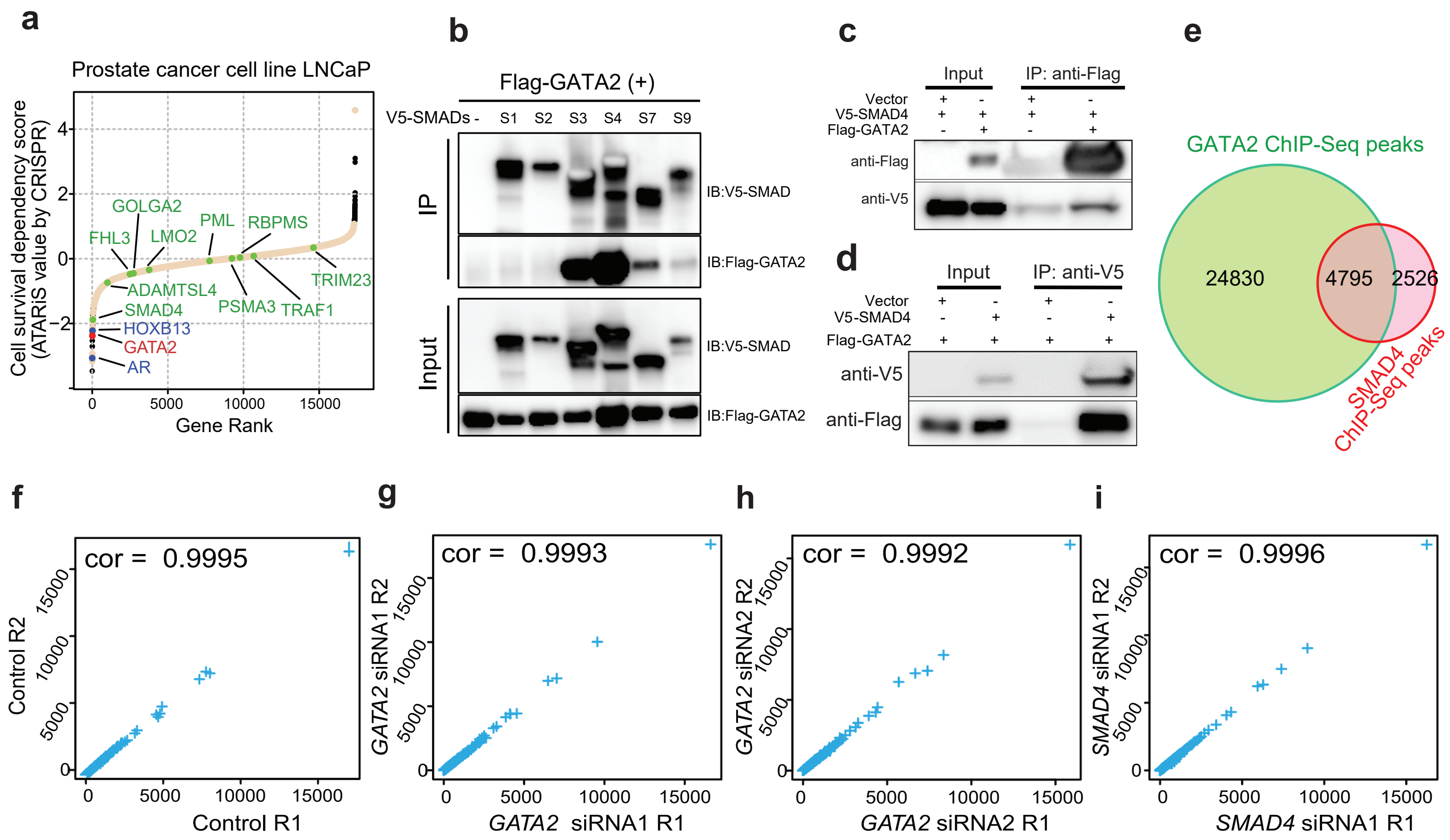

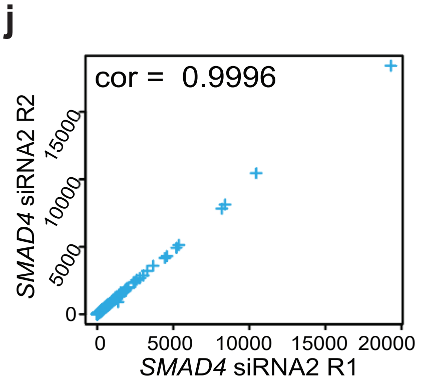

m

GSEA (GATA2)	ES	NES	FDR	Data origin
Proliferation-related gene signatures				
E2F targets	-0.648	-2.715	<0.001	Hallmark
G2M checkpoint	-0.587	-2.463	<0.001	Hallmark
Cell cycle checkpoints	-0.549	-2.412	<0.001	Reactome
Proliferation independent MYC targets	-0.545	-1.967	0.001	Acosta
Cell cycle	-0.502	-1.939	0.003	WikiPathways

GSEA (SMAD4)	ES	NES	FDR	Data origin
Proliferation-related gene signatures				
G2M checkpoint	-0.66	-3.12	<0.001	Hallmark
E2F targets	-0.74	-3.48	<0.001	Hallmark
Cell cycle checkpoints	-0.58	-2.81	<0.001	Reactome
Cell cycle	-0.57	-2.52	<0.001	WikiPathways
Proliferation independent MYC targets	-0.54	-2.24	<0.001	Acosta

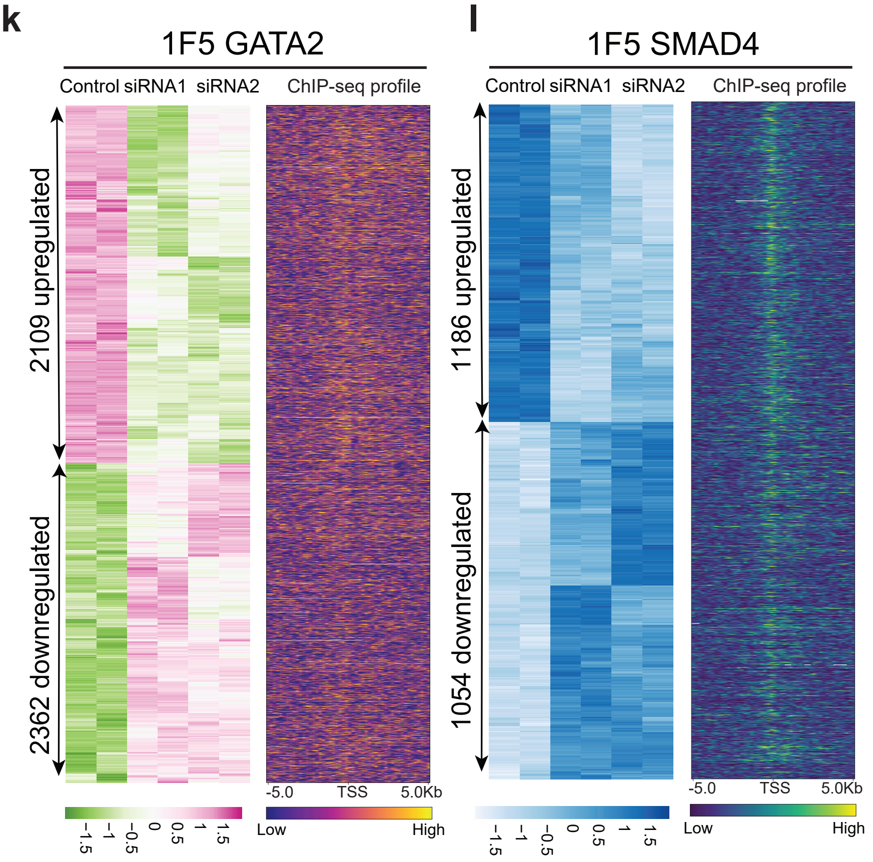

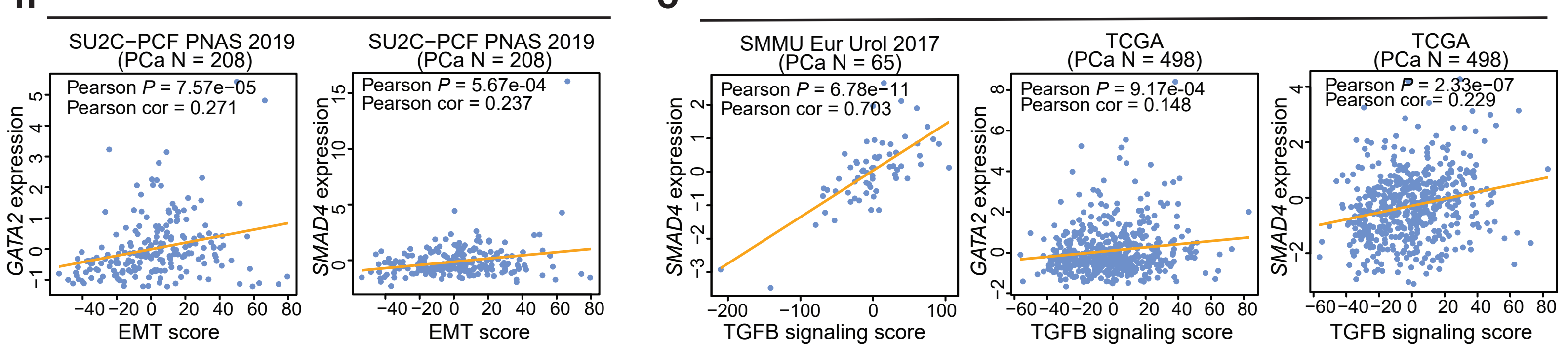

Fig. S4 GATA2 physically interacts and is cooperative with SMAD4 for genome-wide chromatin co-occupancy and co-regulation of PCa genes and cancer metastasis pathways. **a** SMAD4 was identified as an essential gene for cell growth and survival in a genome-wide CRISPR loss-of-function screening in LNCaP cells. Green dots represented the 10 proteins interacting with GATA2 by a comprehensive PPI analysis. Note that only SMAD4 was comparable with GATA2 to be most essential for PCa cell survival. **b** 293T cells were transfected with or without GATA2 together with SMADs. Forty-eight hours later, cell lysates were collected and subjected to co-IP and western blotting analysis. **c-d** Reciprocal immunoprecipitation (IP) from 293T cells ectopically expressing Flag-GATA2 and V5-SMAD4. **e** Venn diagram illustrating the overlap of GATA2 and SMAD4 ChIP-seq binding peaks in 1F5 cells. **f-j** High linear correction between each replicate from GATA2 or SMAD4 RNA-seq in 1F5 cells. **k-l** Heatmaps of genes differentially expressed following GATA2 (**k**) or SMAD4 (**l**) knockdown in 1F5 cells (FDR < 0.1). GATA2 (**k**) or SMAD4 (**l**) ChIP-seq signals in 1F5 cells were plotted for the promoters (TSS ±5 kb) of the genes shown, where yellow indicated higher enrichment. **m** Additional enriched common proliferation-related gene signatures from GATA2 and SMAD4 target gene sets. **n-o** Additional data sets displaying that expression levels of GATA2 or SMAD4 were significantly correlated with the EMT score (**n**) or TGFβ signaling score (**o**) in PCa tumors.

Fig. S5

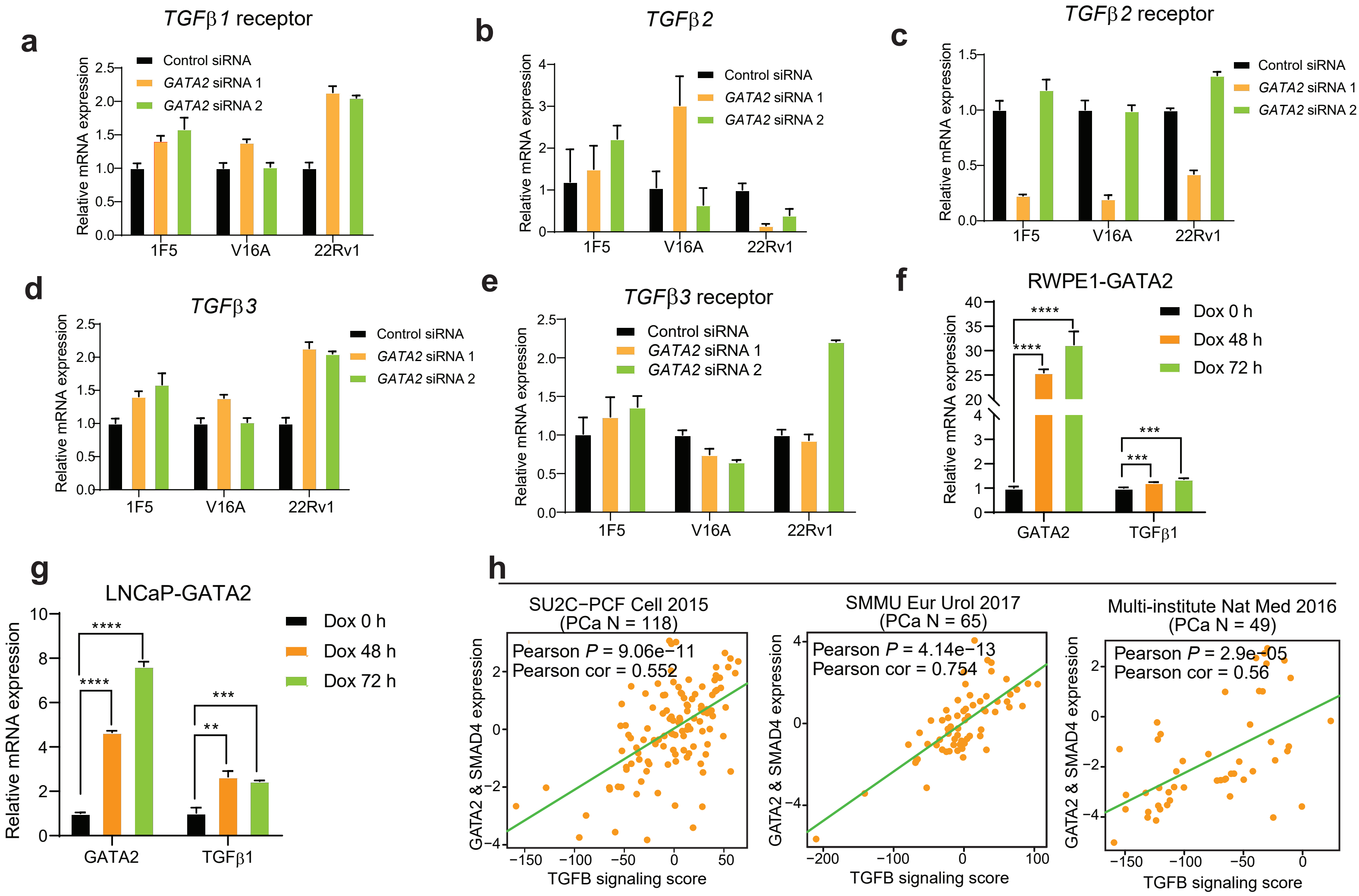

a
TGFβ1 receptor
Relative mRNA expression
Control siRNA
GATA2 siRNA 1
GATA2 siRNA 2
1F5
V16A
22Rv1
b
TGFβ2
c
TGFβ2 receptor
d
TGFβ3
e
TGFβ3 receptor
f
RWPE1-GATA2
Dox 0 h
Dox 48 h
Dox 72 h
GATA2
TGFβ1
g
LNCaP-GATA2
h
SU2C−PCF Cell 2015
(PCa N = 118)
Pearson P = 9.06e−11
Pearson cor = 0.552
SMMU Eur Urol 2017
(PCa N = 65)
Pearson P = 4.14e−13
Pearson cor = 0.754
Multi-institute Nat Med 2016
(PCa N = 49)
Pearson P = 2.9e−05
Pearson cor = 0.56
GATA2 & SMAD4 expression
TGFB signaling score

Fig. S5 GATA2 promotes the expression of *TGFβ1* but not other *TGFβs*. a-e The effect of GATA2 knockdown on the mRNA expression levels of TGFβs as shown in 1F5, V16A and 22Rv1 cells. **f-g** RWPE1-GATA2 and LNCaP-GATA2 cells, respectively, were treated with or without Dox (1μg/mL). Then mRNA expression of *TGFβ1* was determined by qRT-PCR. **h** GATA2 and SMAD4 expression levels positively correlated with TGFβ signaling scores in multiple independent PCa datasets. *P* values assessed by the Pearson's product-moment correlation test. All the error bars are mean s.e.m, n=3 independent experiments. All the error bars represent s.e.m, n=3 technical replicates. $^{**}P<0.001$, $^{***}P<0.001$, $^{****}P<0.0001$, determined by unpaired Student's *t*-test.

Fig. S6

a

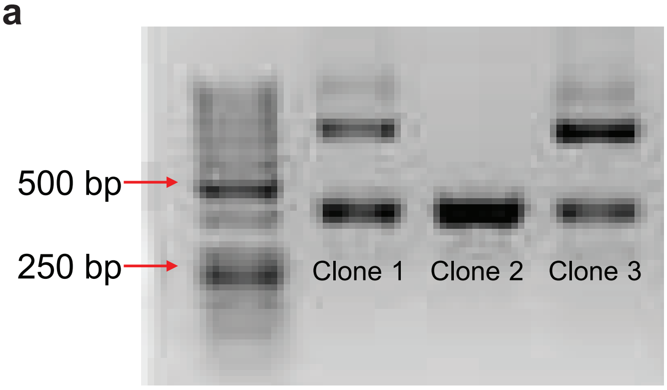
500 bp
250 bp
Clone 1
Clone 2
Clone 3

b

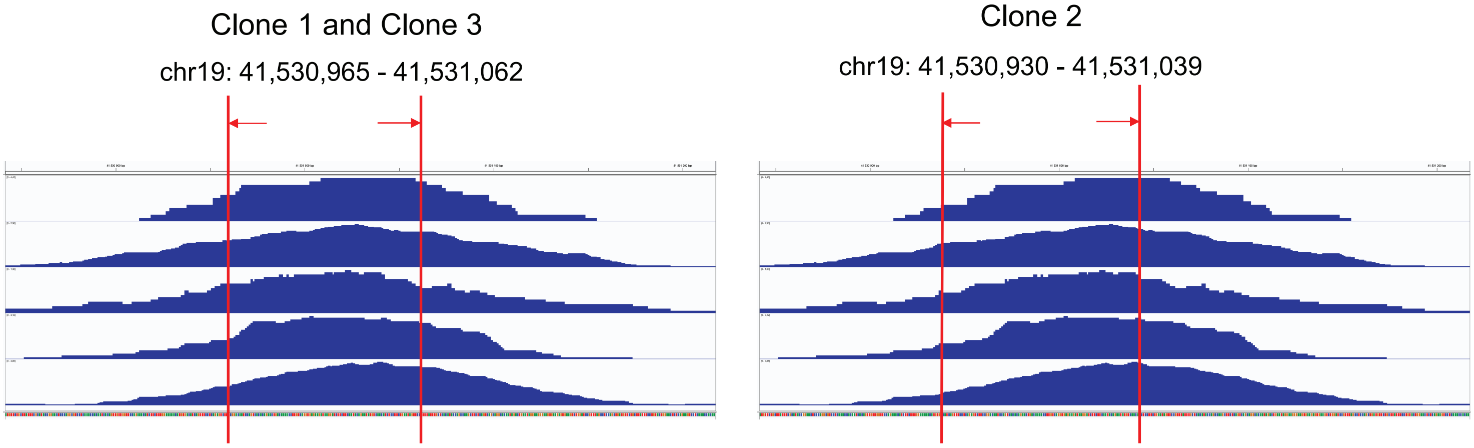
Clone 1 and Clone 3
chr19: 41,530,965 - 41,531,062
Clone 2
chr19: 41,530,930 - 41,531,039

Fig. S6 Deletion of TGFβ1 enhancer by CRISPR/Cas9 genome editing system. a CRISPR/Cas9-mediated deletion of the GATA2-occupied TGFβ1 enhancer. Genomic DNA was subjected to PCR using specific primers and separated by agarose gel in three enhancer knockout clones. The top PCR band indicated the full-length WT PCR amplicons, whereas the lower band indicated the CRISPR-edited PCR amplicons. **b** Gel purification of the genomic DNA from lower bands in (**a**) followed by Sanger Sequencing confirmation.

Fig. S7

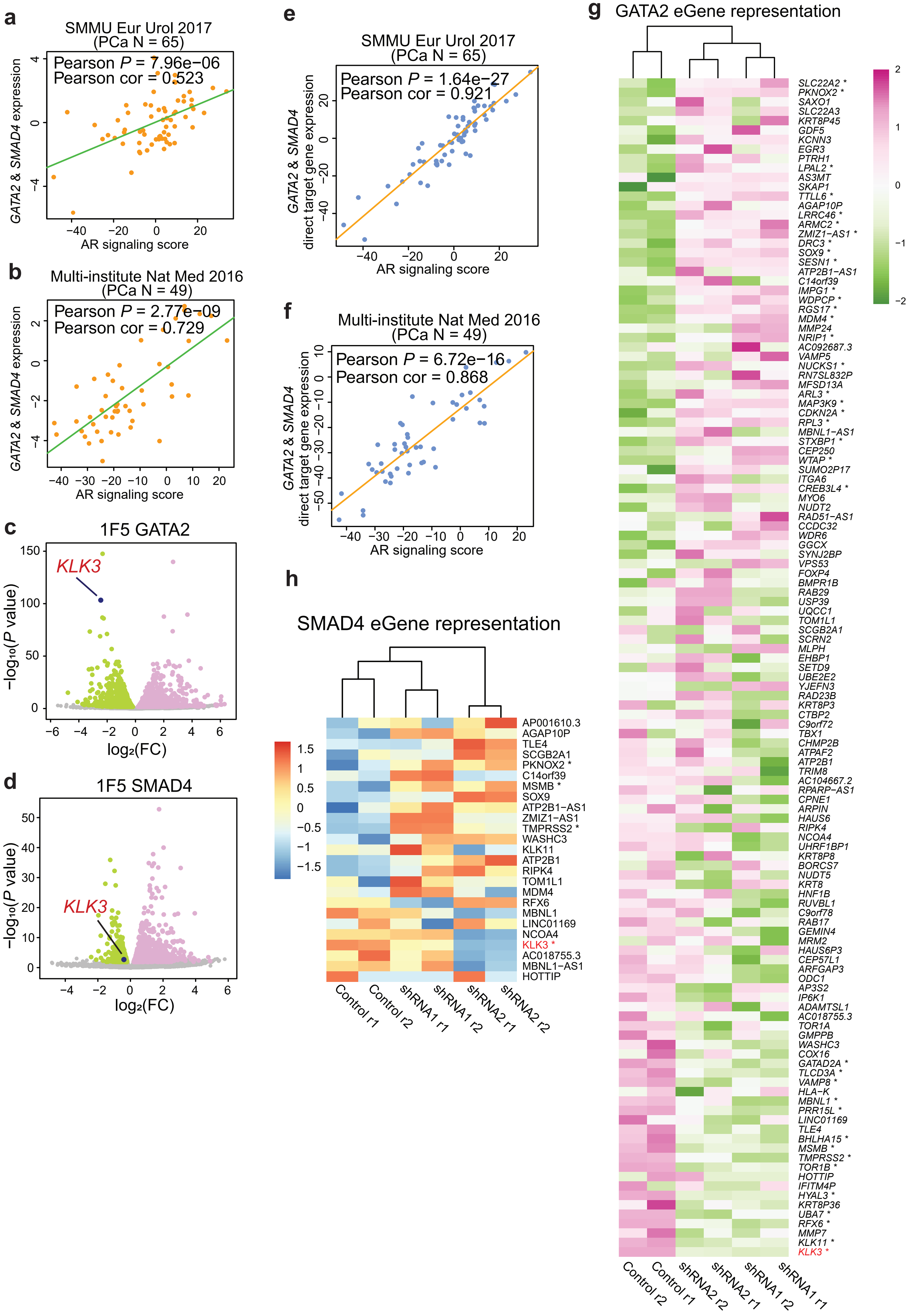
a
SMMU Eur Urol 2017
(PCa N = 65)
Pearson P = 7.96e−06
Pearson cor = 0.523
GATA2 & SMAD4 expression
AR signaling score
b
Multi-institute Nat Med 2016
(PCa N = 49)
Pearson P = 2.77e−09
Pearson cor = 0.729
GATA2 & SMAD4 expression
AR signaling score
c
1F5 GATA2
KLK3
−log10(P value)
log2(FC)
d
1F5 SMAD4
KLK3
−log10(P value)
log2(FC)
e
SMMU Eur Urol 2017
(PCa N = 65)
Pearson P = 1.64e−27
Pearson cor = 0.921
GATA2 & SMAD4 direct target gene expression
AR signaling score
f
Multi-institute Nat Med 2016
(PCa N = 49)
Pearson P = 6.72e−16
Pearson cor = 0.868
GATA2 & SMAD4 direct target gene expression
AR signaling score
h
SMAD4 eGene representation
AP001610.3
AGAP10P
TLE4
SCGB2A1
PKNOX2 *
C14orf39
MSMB *
SOX9
ATP2B1−AS1
ZMIZ1−AS1
TMPRSS2 *
WASHC3
KLK11
ATP2B1
RIPK4
TOM1L1
MDM4
RFX6
MBNL1
LINC01169
NCOA4
KLK3 *
AC018755.3
MBNL1−AS1
HOTTIP
Control r1
Control r2
shRNA1 r1
shRNA1 r2
shRNA2 r1
shRNA2 r2
g
GATA2 eGene representation
SLC22A2 *
PKNOX2 *
SAXO1
SLC22A3
KRT8P45
GDF5
KCNN3
EGR3
PTRH1
LPAL2 *
AS3MT
SKAP1
TTLL6 *
AGAP10P
LRRC46 *
ARMC2 *
ZMIZ1−AS1 *
DRC3 *
SOX9 *
SESN1 *
ATP2B1−AS1
C14orf39
IMPG1 *
WDPCP *
RGS17 *
MDM4 *
MMP24
NRIP1 *
AC092687.3
VAMP5
NUCKS1 *
RN7SL832P
MFSD13A
ARL3 *
MAP3K9 *
CDKN2A *
RPL3 *
MBNL1−AS1
STXBP1 *
CEP250
WTAP *
SUMO2P17
ITGA6
CREB3L4 *
MYO6
NUDT2
RAD51−AS1
CCDC32
WDR6
GGCX
SYNJ2BP
VPS53
FOXP4
BMPR1B
RAB29
USP39
UQCC1
TOM1L1
SCGB2A1
SCRN2
MLPH
EHBP1
SETD9
UBE2E2
YJEFN3
RAD23B
KRT8P3
CTBP2
C9orf72
TBX1
CHMP2B
ATPAF2
ATP2B1
TRIM8
AC104667.2
RPARP−AS1
CPNE1
ARPIN
HAUS6
RIPK4
NCOA4
UHRF1BP1
KRT8P8
BORCS7
NUDT5
KRT8
HNF1B
RUVBL1
C9orf78
RAB17
GEMIN4
MRM2
HAUS6P3
CEP57L1
ARFGAP3
ODC1
AP3S2
IP6K1
ADAMTSL1
AC018755.3
TOR1A
GMPPB
WASHC3
COX16
GATAD2A *
TLCD3A *
VAMP8 *
HLA−K
MBNL1 *
PRR15L *
LINC01169
TLE4
BHLHA15 *
MSMB *
TMPRSS2 *
TOR1B *
HOTTIP
IFITM4P
HYAL3 *
KRT8P36
UBA7 *
RFX6 *
MMP7
KLK11 *
KLK3 *
Control r2
Control r1
shRNA2 r2
shRNA2 r1
shRNA1 r2
shRNA1 r1

Fig. S7 GATA2 co-opts with SMAD4 to regulate AR signaling. a-b Scatter plots showed positive correlations between GATA2 & SMAD4 expression levels and AR signaling score in human PCa. **c-d** Volcano plots demonstrated that *KLK3* was significantly downregulated following knockdown of GATA2 (**c**) or SMAD4 (**d**) in 1F5 cells. Purple or green dots indicated upregulated or downregulated genes, respectively. **e-f** Expression values of GATA2 and SMAD4 joint direct targeting genes positively correlated with AR signaling score in PCa patients. *P* values assessed by the Pearson's product-moment correlation test. **g** Heatmap representation using RNA-seq data upon GATA2 knockdown in 1F5 cells for eQTL genes with the eQTL SNPs enriched in the GATA2 ChIP-seq peaks in multiple PCa cell lines. **h** Heatmap representation using RNA-seq data upon SMAD4 knockdown in 1F5 cells for eQTL genes with the eQTL SNPs enriched in the SMAD4 ChIP-seq peaks in 1F5 cells. Asterisks beside genes in the heatmaps denoted significant differentially expression genes. All the error bars represent s.e.m, n=3 technical replicates. $^{****}P<0.0001$, determined by unpaired student's *t*-test.

Fig. S8

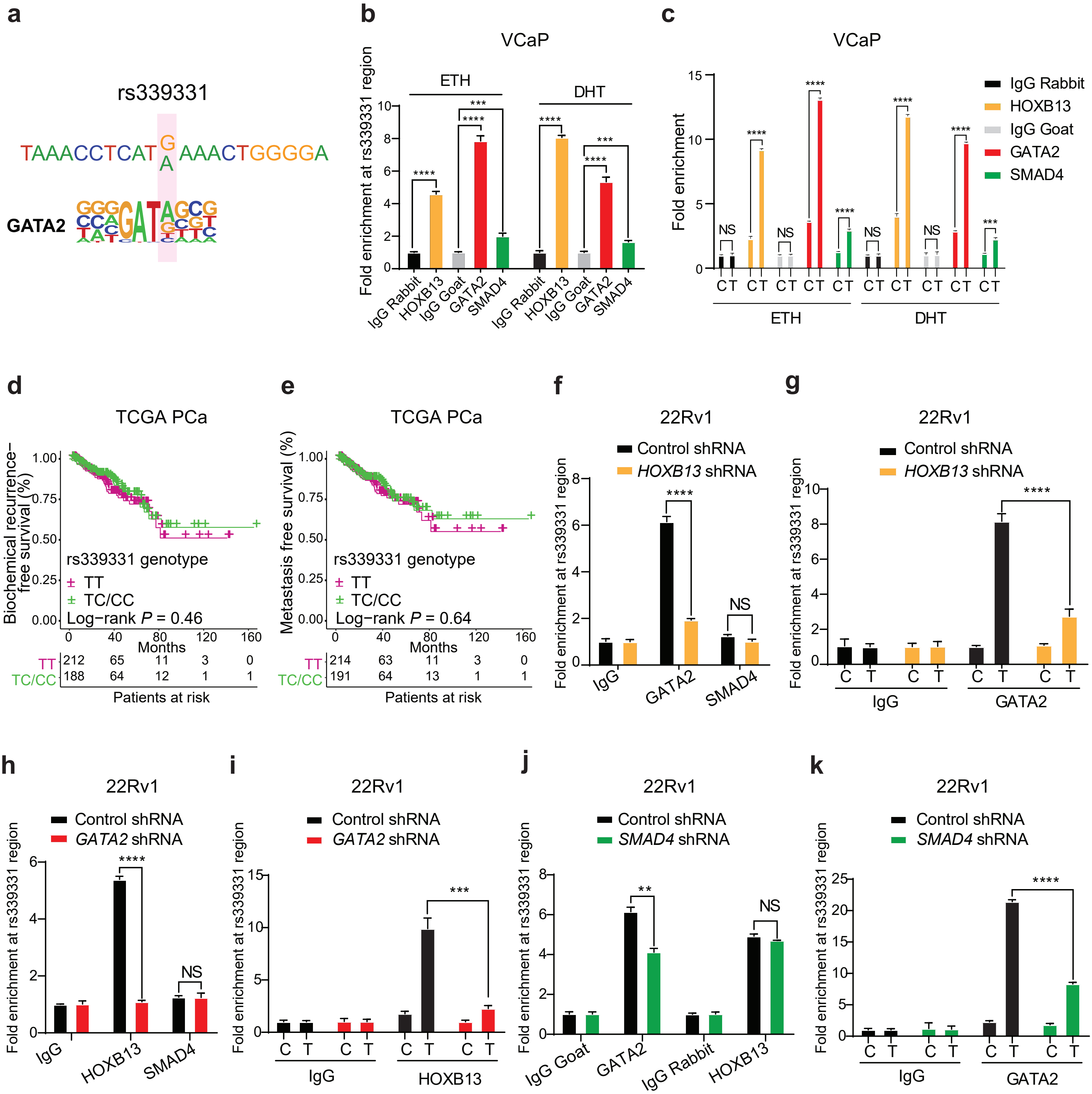

a
rs339331
TAAACCTCAT G/A AAACTGGGGA
GATA2
b
VCaP
ETH
DHT
Fold enrichment at rs339331 region
IgG Rabbit
HOXB13
IgG Goat
GATA2
SMAD4
c
VCaP
Fold enrichment
IgG Rabbit
HOXB13
IgG Goat
GATA2
SMAD4
NS
ETH
DHT
d
TCGA PCa
Biochemical recurrence-free survival (%)
rs339331 genotype
TT
TC/CC
Log−rank P = 0.46
Months
Patients at risk
TT 212 65 11 3 0
TC/CC 188 64 12 1 1
e
TCGA PCa
Metastasis free survival (%)
rs339331 genotype
TT
TC/CC
Log−rank P = 0.64
Months
Patients at risk
TT 214 63 11 3 0
TC/CC 191 64 13 1 1
f
22Rv1
Control shRNA
HOXB13 shRNA
Fold enrichment at rs339331 region
IgG
GATA2
SMAD4
NS
g
22Rv1
Control shRNA
HOXB13 shRNA
IgG
GATA2
h
22Rv1
Control shRNA
GATA2 shRNA
IgG
HOXB13
SMAD4
NS
i
22Rv1
Control shRNA
GATA2 shRNA
IgG
HOXB13
j
22Rv1
Control shRNA
SMAD4 shRNA
IgG Goat
GATA2
IgG Rabbit
HOXB13
NS
k
22Rv1
Control shRNA
SMAD4 shRNA
IgG
GATA2

Fig. S8 GATA2 and SMAD4 indicate a tendency of binding to the PCa-associated risk T allele at rs339331/6q22. a Representation of rs339331 located within the GATA2 DNA-binding motif in the reverse strand. **b** ChIP-qPCR for GATA2 and SMAD4 chromatin binding at the rs339331-containing region upon ETH or DHT treatment for 24h in VCaP cells. HOXB13 as a positive control. **c** GATA2 and SMAD4 favor binding to the T risk allele than C at rs339331 as determined by ChIP followed by AS-qPCR in ETH or DHT treated VCaP cells. **d-e** rs339331 could not stratify PCa patients with biochemical recurrence (**d**) or metastasis (**e**). *P* values assessed by the log-rank test. **f-g** Knockdown of HOXB13 decreased the capacity of GATA2, but not SMAD4, binding to rs339331 and its T allele. **h-i** Knockdown of GATA2 alleviated the capability of HOXB13, but not SMAD4, binding to rs339331 and its T allele. **j-k** Knockdown of SMAD4 reduced the potential of GATA2, but not HOXB13, binding to rs339331 and its T allele. $^{***}P<0.001$, $^{****}P<0.0001$, determined by unpaired student's *t*-test. N.S: Non-significant.

Fig. S9

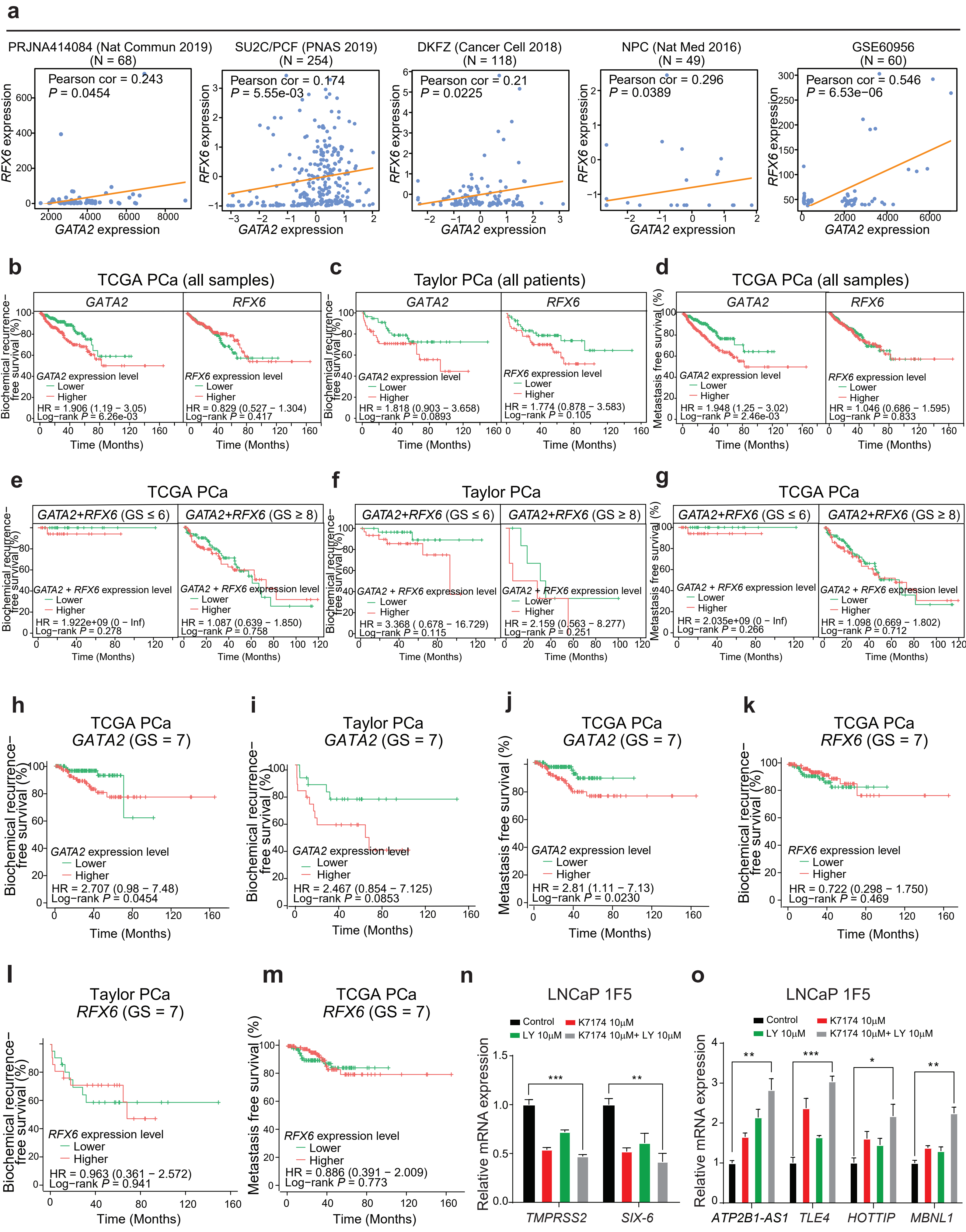

a
PRJNA414084 (Nat Commun 2019) (N = 68)
Pearson cor = 0.243
P = 0.0454
SU2C/PCF (PNAS 2019) (N = 254)
Pearson cor = 0.174
P = 5.55e-03
DKFZ (Cancer Cell 2018) (N = 118)
Pearson cor = 0.21
P = 0.0225
NPC (Nat Med 2016) (N = 49)
Pearson cor = 0.296
P = 0.0389
GSE60956 (N = 60)
Pearson cor = 0.546
P = 6.53e−06
RFX6 expression
GATA2 expression
b
TCGA PCa (all samples)
GATA2
RFX6
GATA2 expression level
RFX6 expression level
Lower
Higher
HR = 1.906 (1.19 − 3.05)
Log−rank P = 6.26e-03
HR = 0.829 (0.527 − 1.304)
Log−rank P = 0.417
Biochemical recurrence-free survival (%)
Time (Months)
c
Taylor PCa (all patients)
HR = 1.818 (0.903 − 3.658)
Log−rank P = 0.0893
HR = 1.774 (0.878 − 3.583)
Log−rank P = 0.105
d
TCGA PCa (all samples)
Metastasis free survival (%)
HR = 1.948 (1.25 − 3.02)
Log−rank P = 2.46e-03
HR = 1.046 (0.686 − 1.595)
Log−rank P = 0.833
e
TCGA PCa
GATA2+RFX6 (GS ≤ 6)
GATA2+RFX6 (GS ≥ 8)
GATA2 + RFX6 expression level
HR = 1.922e+09 (0 − Inf)
Log−rank P = 0.278
HR = 1.087 (0.639 − 1.850)
Log−rank P = 0.758
f
Taylor PCa
HR = 3.368 (0.678 − 16.729)
Log−rank P = 0.115
HR = 2.159 (0.563 − 8.277)
Log−rank P = 0.251
g
TCGA PCa
HR = 2.035e+09 (0 − Inf)
Log−rank P = 0.266
HR = 1.098 (0.669 − 1.802)
Log−rank P = 0.712
h
TCGA PCa
GATA2 (GS = 7)
HR = 2.707 (0.98 − 7.48)
Log−rank P = 0.0454
i
Taylor PCa
GATA2 (GS = 7)
HR = 2.467 (0.854 − 7.125)
Log−rank P = 0.0853
j
TCGA PCa
GATA2 (GS = 7)
HR = 2.81 (1.11 − 7.13)
Log−rank P = 0.0230
k
TCGA PCa
RFX6 (GS = 7)
HR = 0.722 (0.298 − 1.750)
Log−rank P = 0.469
l
Taylor PCa
RFX6 (GS = 7)
HR = 0.963 (0.361 − 2.572)
Log−rank P = 0.941
m
TCGA PCa
RFX6 (GS = 7)
HR = 0.886 (0.391 − 2.009)
Log−rank P = 0.773
n
LNCaP 1F5
Control
K7174 10μM
LY 10μM
K7174 10μM+ LY 10μM
Relative mRNA expression
***
**
TMPRSS2
SIX-6
o
LNCaP 1F5
**
***
*
**
ATP2B1-AS1
TLE4
HOTTIP
MBNL1

Fig. S9 The synergistic effects of GATA2 and RFX6 expression levels in PCa progression and prognosis. a Expression levels of RFX6 positively correlated with GATA2 in multiple independent PCa cohorts. The gene expression level in the PRJNA414084 dataset was measured by the RNA-seq and normalized by the median of ratios method from the DESeq2. **b-d** Kaplan Meier plots showed the values of biochemical recurrence and metastasis of PCa patients with high GATA2 or RFX6 expression level in two independent cohorts. **e-g** Joint expression levels of GATA2 and SMAD4 showed failure in stratifying PCa patients with lower (Gleason score 6) or higher risks (Gleason score >=8). *P* values assessed by log-rank test. **h-m** GATA2 or RFX6 mRNA levels alone indicated less prognostic value in PCa patient group with an intermediate risk of Gleason Score 7 when compared to the synergistic expression levels of GATA2 and RFX6 (see **Fig. 8q-r**). PCa samples with RFX6 deep loss were excluded in the analysis. Patients were stratified by the median value of RFX6 and GATA2 expression levels. *P* values assessed by log-rank test. **n-o** The combination effect of TGFβ signaling inhibitor and AR antagonist on the expression of the GATA2 and SMAD4 common targeting eQTL genes. $^{*}P<0.05$, $^{**}P<0.001$, $^{***}P<0.001$, determined by unpaired student's *t*-test.
